# Activation of 1-Aminocyclopropane-1-Carboxylic Acid Synthases Sets Stomatal Density and Clustered Ratio on Leaf Epidermis of *Arabidopsis* in Response to Drought

**DOI:** 10.3389/fpls.2021.758785

**Published:** 2021-12-06

**Authors:** Ming-zhu Jia, Ling-yun Liu, Chen Geng, Jing Jiang

**Affiliations:** State Key Laboratory of Cotton Biology, State Key Laboratory of Crop Stress Adaptation and Improvement, College of Life Sciences, Henan University, Kaifeng, China

**Keywords:** ACC, ACS, Ca^2+^, stomatal density, stomatal cluster, stomatal space, drought

## Abstract

The adjustment of stomatal density and clustered ratio on the epidermis is the important strategy for plants to respond to drought, because the stoma-based water loss is directly related to plant growth and survival under drought conditions. But the relevant adjustment mechanism still needs to be explored. 1-Aminocyclopropane-1-carboxylate (ACC) is disclosed to promote stomatal development, while *in vivo* ACC levels depend on activation of ACC synthase (ACS) family members. Based on the findings of ACS expression involving in drought response and several ACS activity inhibitors reducing stomatal density and cluster in drought response, here we examined how ACS activation is involved in the establishment of stomatal density and cluster on the epidermis under drought conditions. Preliminary data indicated that activation of ACS2 and/or ACS6 (ACS2/6) increased stomatal density and clustered ratio on the *Arabidopsis* leaf epidermis by accumulating ACC under moderate drought, and raised the survival risk of seedlings under escalated drought. Further exploration indicated that, in *Arabidopsis* seedlings stressed by drought, the transcription factor SPEECHLESS (SPCH), the initiator of stomatal development, activates ACS2/6 expression and ACC production; and that ACC accumulation induces Ca^2+^ deficiency in stomatal lineage; this deficiency inactivates a subtilisin-like protease STOMATAL DENSITY AND DISTRIBUTION 1 (SDD1) by stabilizing the inhibition of the transcription factor GT-2 Like 1 (GTL1) on *SDD1* expression, resulting in an increases of stomatal density and cluster ratio on the leaf epidermis. This work provides a novel evidence that ACS2/6 activation plays a key role in the establishment of stomatal density and cluster on the leaf epidermis of *Arabidopsis* in response to drought.

## Introduction

Stomata are pore structures surrounded by guard cells (GCs) on the leaf epidermis that regulate the exchange of gases (i.e., H_2_O, CO_2_, or O_2_) between plants and the environment (Acharya and Assmann, 2009; Zoulias et al., 2018). In evolution from aquatic to terrestrial, plants had generated stomata on epidermis of aerial part to facilitate transpiration, and to guarantee plant survival and life on land (Croxdale, 2000; van Veen and Sasidharan, 2021). For terrestrial plant, stomata can sense environmental water status, especially water deficit or drought, and regulate water loss from plant (Acharya and Assmann, 2009; Zoulias et al., 2018). The ability of stomata to regulate water loss is generally estimated from stomatal density (number of stomata per unit leaf area), stomatal index (ratio of stomatal number to sum of epidermal cells and stomata), and pattern (whether stomata are distributed singly or in clusters). Evidences show that plants accurately set the number of epidermal cells between the starting position for development of new stoma and the preexisting stomata, that is, set the stomatal space under certain conditions (Croxdale, 2000; Hamanishi et al., 2012; Zoulias et al., 2018). That is to say, stomatal density and clustered ratio are the results of stomatal space setting; for example, stomatal cluster is formed by directly contacting stomata with no intervening epidermal cell, or, alternatively, zero-space establishment (Von Groll et al., 2002; Acharya and Assmann, 2009; Zoulias et al., 2018). The establishment of stomatal space is, therefore, an important aspect of plant growth and survival under drought conditions (Hepworth et al., 2015). However, the setting mechanism of stomatal space needs to be studied.

The regulation of stomatal development, which undergirds stomatal space setting, has been extensively investigated. Studies on the model plant *Arabidopsis thaliana* (L.) have shown that stomatal development includes a series of epidermal cell divisions, in which several basic helix-loop-helix transcription factors, such as SPCH, MUTE, and FAMA, are involved in this process. SPCH initiates stomatal development to transform meristemoid mother cells (MMCs) into a meristemoids and a sister cell; MUTE converts a sister cell into the guard mother cells (GMCs); then FAMA drives GMCs to form a stoma with differentiated GCs (Hamanishi et al., 2012; Zoulias et al., 2018). These findings show clearly that SPCH dominates stomatal development. This view is better interpreted, for example, the loss-of-function *spch-1* or *spch-3* homozygous mutant do not produce any stomata (MacAlister et al., 2007; Pillitteri et al., 2007; Han and Torii, 2016). Furthermore, SPCH expression is known to implicate the establishment of stomatal density (Tripathi et al., 2016; Zoulias et al., 2018). However, it is still unclear whether and how SPCH-dependent stomatal development affects stomatal space setting.

Evidences suggest that the subtilisin-like protease STOMATAL DENSITY AND DISTRIBUTION 1 (SDD1) participates in the establishment of stomatal density and clustered ratio on leaf epidermis (Berger and Altmann, 2000; Casson and Gray, 2008; Serna, 2009; Zoulias et al., 2018), as evidenced by the following: a loss-of-function *sdd1-1* mutant showed a two- to four-fold increase in stomatal density and clusters in all aerial parts, whereas transgenic *SDD1-*overexpressing plants exhibited a two- to three-fold decrease in stomatal density and arrested stomata (Berger and Altmann, 2000; Von Groll et al., 2002). In line with these findings, *SDD1*-overexpressing plants displayed diminished transpiration because of a ∼25% reduction in abaxial stomatal density or clusters (Yoo et al., 2010). Evidently, SDD1 activity specifically increases stomatal density and stomatal clustering ratio, or reduces stomatal space. But the regulatory mechanism of SDD1 activity has been uncovered. Significantly, the trihelix transcription factor GT-2 LIKE 1 (GTL1) binds to the promoter of the *SDD1* gene and inhibits its expression (Yoo et al., 2010; Weng et al., 2012; Virdi et al., 2015). This inhibition can be relieved by Ca^2+^ increase because Ca^2+^-loaded calmodulin (Ca^2+^-CaM) destabilizes the docking of GTL1 protein to the *SDD1* promoter (Yoo et al., 2019). Clearly, elevated Ca^2+^ levels increase SDD1 activity but decrease stomatal density and clustered ratio. Nevertheless, it remains unclear how SPCH-dependent stomatal individual development is integrated with SDD1-controlled stomatal space setting.

The non-proteinogenic amino acid 1-aminocyclopropane-1-carboxylate (ACC) has recently been shown to independently promote stomatal generation by facilitating the differentiation of GMCs into GCs in *Arabidopsis* leaves (Yin et al., 2019). Unexpectedly, ethylene is not involved in this process (Yin et al., 2019), even though ACC is the precursor of ethylene (Bleecker and Kende, 2000). In fact, ACC is known to be involved in stomatal development and space setting (Acharya and Assmann, 2009). For example, ACC treatments increased the number of stomata by ∼33% on the hypocotyl (Saibo et al., 2003) or cotyledon epidermis (Serna and Fenoll, 1996) in *Arabidopsis*, and also induced stomatal clusters (Serna and Fenoll, 1996; Berger and Altmann, 2000). The production of ACC *in vivo* depends on the activity of ACC synthase (ACS), which converts *S*-adenosylmethionine to ACC (Bleecker and Kende, 2000). Various pieces of experimental evidence strongly suggest that ACS activity is an important mediator of stomata development. For example, the inhibitors of ACS activity, such as aminoethoxyvinylglycine (AVG), were shown to significantly reduce the frequency of stomatal appearance (Serna and Fenoll, 1996; Saibo et al., 2003; Yin et al., 2019), which is the prerequisite for stomatal space setting. It is known that ACS is encoded by a multi-gene family (Bleecker and Kende, 2000), and that the activity of ACS family members is unique, overlapping, and spatiotemporally specific (Tsuchisaka and Theologis, 2004; Tsuchisaka et al., 2009). The *Arabidopsis* genome contains nine *ACS* genes (*ACS1, ACS2, ACS4–9*, and *ACS11*) that encode authentic enzymes (Tsuchisaka et al., 2009). Few evidences imply that ACS activity may be involved in the stomata-based drought response, for example, the expression of *ACS* genes is induced by drought in *Arabidopsis* (Dubois et al., 2017, 2018), and chromatin immunoprecipitation assays indicate that SPCH may regulate the transcription activity of *ACS2* and *ACS6* genes (Lau et al., 2014). Nevertheless, further evidence needs to be provided that how SPCH directs ACS2/6 activity during stomatal developing and spacing.

In this study, we explored the specific involvement of ACS2/6 activity in the drought tolerance of *Arabidopsis* seedlings. Our results revealed that the T-DNA insertion mutants *acs2-1*, *acs6-1*, and *acs2-1acs6-1* are more tolerant to drought than is the wild-type (WT) control. Subsequent research on the underlying mechanism indicated that SPCH activates the expression of *ACS2* and/or *ACS6* by directly binding to their promoters. ACS2/6-dependent ACC accumulation triggers a Ca^2+^ shortage in stomatal lineage cells, and thus stabilized the inhibition of the transcription factor GT-2 Like 1 (GTL1) on *SDD1* expression. Stomatal density and cluster on the leaf epidermis are thereby increased, leading to increased seedling wilting and even death under intensified drought.

## Materials and Methods

### Plant Materials and Growth Conditions

*Arabidopsis thaliana* (Columbia-0 ecotype) was used as WT. The different *ACS2* expression lines, including mutant *acs2-1* (CS16564) with a T-DNA insertion, *ACS2*-complementation (*ACS2*/*acs2-1*), *ACS2*-overexpression (*ACS2*-OE), and *pACS2*::*ACS2-GUS* lines, have been described previously (Han et al., 2019). The T-DNA insertion mutant *acs6-1* (CS16569) was obtained from the Arabidopsis Biological Resource Center (United States). The double mutant *acs2-1acs6-1* was created by crossing *acs2-1* with *acs6-1*. Seeds of *spch-3* mutant with a T-DNA insertion were a friendly gift from Professor Sui-wen Hou (MOE Key Laboratory of Cell Activities and Stress Adaptations, Lanzhou, China) which were described in MacAlister et al. (2007). These homozygotes with T-DNA insertion were screened according to the method provided by the Salk Institute^1^. Seeds of the point mutant *spch-1* was a friendly gift from Professor Xiao-lan Chen (School of Life Sciences, Yunnan University, China) which were created by MacAlister et al. (2007), and was identified by PCR amplification and sequencing of the fragment containing the mutation site. *spch-1acs2-1acs6-1* were generated by genetic crossing with reference to Han et al. (2018). All primers used in this study are listed in the Supplementary Table 1.

Seeds of the transgenic *pSPCH*::*SPCH-GFP* line (created by Fred Sack, University of British Columbia) were a friendly gift from Professor Xiao-lan Chen (School of Life Sciences, Yunnan University, China), and GFP expression was detected by hygromycin screening and measurement of fluorescence in leaves. The seeds of Ca^2+^ sensor NES-YC3.6-expressing line were kindly gifted by Professor Jörg Kudla (Molecular Genetics and Cell Biology of Plants, University of Munich, Germany). NES-YC3.6-expressing *acs2-1acs6-1* line was created by crossing *acs2-1acs6-1* with NES-YC3.6-expressing WT plants. Progeny were selected on kanamycin-containing medium and by measuring fluorescence in leaves. All F_3_ progeny meeting the requirements were used in subsequent experiments.

All seeds were collected and stored under the same conditions. Prior to experiments, seeds were surface-sterilized and sown on Murashige-Skoog medium. After 3 days at 4°C in darkness, plates were transferred to a greenhouse (21 ± 2°C, 70% humidity, 100 μmol m^–2^ s^–1^ light intensity, and a 16-h light/8-h dark photoperiod). After germination and growth for 7 days, young seedlings were transplanted into water-saturated soil. Watering was halted according to the requirements of each specific drought treatment described in this paper.

### Creation of Transgenic Plants

To generate *ACS6*- and *SPCH*-overexpression lines, the full-length coding sequence (CDS) of *ACS6* or *SPCH* was amplified and cloned into the pSUPER 1300 vector. Each construct was then introduced into *Agrobacterium* strain GV3101 and transformed into the target plants by floral infiltration. The same method was used to generate the transformants described below. To generate *ACS6*-complementation (*ACS6*/*acs6-1*) lines, the promoter and CDS of *ACS6* were cloned into a pCAMBIA1300 vector, which was transformed into *acs6-1* plants. To generate *pACS6*::*ACS6-GUS* lines, the *ACS6* promoter fragment and full-length CDS were cloned into the promoter-less β-glucuronidase (GUS) expression vector pCAMBIA1391, which was then transformed into WT. To generate *pSDD1*::*SDD1-GFP* lines, the promoter fragment and CDS of *SDD1* were cloned into a pCAMBIA1300 vector, which was then introduced into *acs2-1acs6-1* and WT. The T_1_ transgenic plants were selected on hygromycin-containing medium, and the T_3_ progeny were used for subsequent experiments.

### Water Loss Assay

True leaves were collected from 28-day-old plants following previously described methods (Xie et al., 2019). The fresh weight of leaves was determined immediately. Leaves of five plants per line were weighed hourly on an electronic balance (Sartorius, Germany) at room temperature (23°C). Water loss was calculated using the following formula: [(W1–W2)/W1] × 100%, where W1 is the initial leaf fresh weight, and W2 is the leaf weight at a given time point.

### Evaluation of Stomatal Density, Stomatal Index, and Rate of Stomatal Clustering

Stomatal density, stomatal index and clustering ratio were determined according to previously described methods (de Marcos et al., 2017; Qi et al., 2019). The sixth fully expanded rosette leaves (count up from cotyledons) were used for analyzing the stomatal phenotype of 28-day-old seedlings. Strips were peeled from leaf abaxial epidermis, fixed on a slide, and photographed under a differential contrast interference microscope (LSM710, Zeiss, Germany). Images were acquired under the 20× objective (0.18 mm^2^). Randomly selected images are shown in figures.

For analyses of stomatal density, index and clustering rate, 25 plants per line per plant were examined. In all counts, a stoma was considered to have a pair of complete guard cells. The calculation formula involved is as follows: Stomatal density = stomatal number/area (mm^2^); Stomatal index = (number of stomata)/(number of epidermal cells + number of stomata) × 100%; The rate of clustered stomata = number of clusters/(number of stomata + number of clusters) × 100%.

### RNA Extraction and Quantitative Real-Time Polymerase Chain Reaction Analyses

Total RNA was extracted using a plant RNA MIDI kit (Life-Feng, Shanghai, China). First-strand complementary DNA (cDNA) was synthesized with a Reverse Transcription system (Toyobo, Osaka, Japan) and was used as the template for quantitative real-time polymerase chain reaction (RT-qPCR) analyses along with 2 × SYBR Green I master mix (Vazyme, Nanjing, China). The RT-qPCR analyses were performed on a Roche 480 real-time PCR system (Roche, Mannheim, Germany). The RNA levels were calculated as described by Livak and Schmittgen (2001). The reference gene was *ACTIN8* (AT1G49240).

### β-Glucuronidase Staining

Leaves excised from 21-day-old plants were incubated overnight in darkness at 37°C in GUS staining solution (0.1 M sodium phosphate buffer, pH 7.0; 0.05 mM K_3_[Fe(CN)_6_]; 0.05 mM K_4_[Fe(CN)_6_]; 1 mg ml^–1^ X-Gluc (Sigma, United States); and 0.1% Triton X-100). After staining, leaves were de-stained with 75% (v/v) ethanol until the chlorophyll was completely removed, and then were photographed using a digital camera (Canon 760D). Representative photographs are shown in figures.

### Measurement of 1-Aminocyclopropane-1-Carboxylate Content

Leaves from the same line of 21-day-old plants were collected and ground into a powder. A 0.1-mg aliquot of powdered sample was transferred into an Eppendorf tube along with 1 ml ultrapure water. To completely extract ACC from leaf tissue, the sample was further fragmented using an ultrasonic crusher (Branson, Danbury, CT, United States). The supernatant was collected, the pH was adjusted to <4, and impurities were removed using 1 ml chloroform. The supernatant was then passed through a column containing C18 adsorbent (Oasis MCX, 30 μm, 3 cc/60 mg, Waters, Milford, MA, United States). The column was eluted with 1 M ammonia in water, with chromatographic methanol as the solvent. The eluent was evaporated to dryness in a Concentrator Plus evaporator (Eppendorf, Hamburg, Germany) under vacuum at 30°C and then re-suspended in solution (chromatographic methanol: 0.1% (v/v) acetic acid, 1:9). Samples were analyzed using an Applied Biosystems MDS SCIEX 4000 QTRAP liquid chromatography-tandem mass spectrometry system (AB Sciex, Foster City, CA, United States). Standard ACC (Sigma-Aldrich, Steinheim, Germany) was used for the quantitative analysis.

### Protein Extraction and Western Blotting

Leaves of 21-day-old *pSDD1*::*SDD1-GFP* transgenic plants were collected according to the experimental requirements and ground into a powder. Powdered samples were transferred to RIPA lysis buffer (Boster Biotechnology, Wuhan, China) and micro-centrifuged at 16,000 × *g* for 15 min at 4°C. The concentration of crude protein in the supernatant was determined using a NanoDrop 2000 (Thermo Scientific Wilmington, DE, United States). The crude protein was separated by 12% SDS-PAGE and then transferred to a nitrocellulose filter membrane (Millipore, Billerica, MA, United States) using a Trans-Blot Semi-Dry transfer cell (Bio-Rad, Hercules, CA, United States). The membrane was then incubated at room temperature for 1–2 h in blocking solution before incubation with anti-GFP mouse monoclonal antibodies (1:10,000; Proteintech, Chicago, IL, United States) for 2 h at room temperature. The membrane was subjected to three 10-min washes with TBST and then incubated overnight at 4°C with horseradish peroxidase-conjugated secondary antibody (Proteintech). Protein bands were detected using a BeyoECL Plus kit (Beyotime, Shanghai, China) and then visualized using a Fusion FX7 Spectra system (Vilber Lourmat, Marne-la-Vallée, France). An anti-GAPDH antibody (1:5000; Proteintech) was used as the loading control.

### Chromatin Immunoprecipitation Analyses

Immature leaves collected from *pSPCH::SPCH-GFP* of 21-day-old plants were cross-linked using 1% formaldehyde under vacuum for 10 min according to the EZ-ChIP chromatin IP kit protocol (Thermo Scientific). After washing with phosphate-buffered saline solution, leaves were ground in liquid nitrogen and then suspended in SDS lysis buffer containing protease inhibitor cocktail. The DNA of *SPCH* was sheared into small fragments (300–500 bp). The sheared chromatin was chromatin immunoprecipitation with GFP antibodies (Proteintech) overnight at 4°C. The ChIP DNA products were analyzed by RT-qPCR using three pairs of primers synthesized to amplify approximately 200-bp DNA fragments of the promoter region of *ACS2* or *ACS6*, which were used in the ChIP analysis. Primers annealing to promoter regions of two *Arabidopsis* genes lacking an SPCH binding site were used as negative controls. An unrelated DNA sequence from the *ACTIN8* gene was used as an internal control.

### Transient Transcription Dual-Luciferase Assays

Detection was performed according to previously described methods (Bao et al., 2014). The 2400-bp promoter sequence of *ACS2* was divided into three fragments (−1 to −1000, −900 to −1600, and −1500 to −2400 bp). The 2600-bp promoter sequence of *ACS6* was also divided into three fragments (−1 to −1000, −900 to −2000, and −1900 to −2600 bp). Each fragment was cloned into pGreen II 0800-Luc to construct the corresponding reporter plasmid. The coding sequence of *Arabidopsis SPCH* was cloned into pGreenII 62-SK to construct the 35S-SPCH effector plasmid. The *Agrobacterium* strain GV3101 (pSoup-p19) was incubated in yeast mannitol medium and finally re-suspended in buffer to a final concentration of OD_600_ = 1.0. Equal amounts of different combined bacterial suspensions were infiltrated into young leaves of tobacco plants using a needleless syringe. After 3 days, the infected leaves were sprayed with D-luciferin (sodium salt) (Yeasen, Shanghai, China) and placed in darkness for 5 min. Firefly luciferase (LUC) signals were then detected using the NightSHADE system (LB 985, Berthold Technologies, Bad Wildbad, Germany). The ratio of LUC activity to Renilla luciferase (REN) activity was measured using a Dual-Luciferase Reporter Gene Assay kit (Solarbio, Beijing, China). Briefly, the tobacco leaves were ground in liquid nitrogen, and the extract was incubated in a low-temperature buffer. The LUC/REN ratio was measured using an enzyme standard instrument (Tecan, Männedorf, Switzerland).

### Monitoring of Ca^2+^ Levels in Stomatal Lineage Cells

The Ca^2+^ levels in stomatal lineage cells were monitored according to Krebs et al. (2012). Immature leaves of *Arabidopsis* seedlings expressing the fluorescence resonance energy transfer (FRET)-based Ca^2+^ sensor NES-YC3.6 (Nagai et al., 2004; Krebs et al., 2012) were collected from the same position. During confocal laser scanning, strips were peeled from leaf abaxial epidermis and then fixed on a slide on the loading platform. The relative fluorescence intensity of YC3.6 protein was recorded under a Nikon A1 Plus laser scanning confocal microscope (Nikon, Tokyo, Japan) with the following scanning parameters: image dimension = 1024 × 1024, pinhole radius = 38.31 μm, scanning speed = 0.25, zoom = 3×, objective = 60× (water), numerical aperture = 1.27), plan apochromat objective, power = 6% (445 nm solid laser). Images were acquired every 5s. Emissions from cyan fluorescent protein (CFP; 465–499 nm) and FRET-dependent cpVenus (525–555 nm) in stomatal lineage were detected simultaneously. The cpVenus/CFP emission ratio was analyzed using NIS-Elements AR software.

### Statistical Analysis

All experiments were independently repeated using three biological replicates and three technical replicates at least. Statistical analysis comparing two means were performed using the two-way ANOVA or Student’s *t*-test [*P*-values < 0.05 (*) and <0.01 (^**^) were considered to correspond to significant and extremely significant differences, respectively].

## Results

### ACS2/6 Activation and 1-Aminocyclopropane-1-Carboxylate Accumulation Facilitated Water Evaporation From Leaves in Response to Drought Treatment

Studies have shown that the activity of ACS2 in rice (Zhang et al., 2013) and ACS6 in maize (Young et al., 2004) regulates seedling sensitivity to drought, and that drought induces ACS2/6 activation and ACC accumulation in *Arabidopsis* (Catalá et al., 2014; Dubois et al., 2018). Therefore, we examined the possible roles of ACS2 and ACS6 in the drought response of *Arabidopsis* seedlings.

**FIGURE 1:**
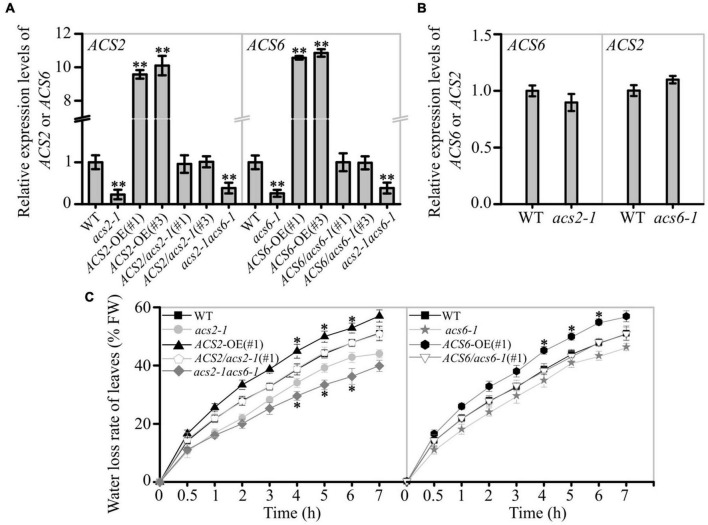
Effect of ACS2/6 expression on water loss from leaves of *Arabidopsis* seedlings. **(A)** RT-qPCR analysis of *ACS2* and *ACS6* mRNA levels in various lines, including the WT; loss-of-function mutants *acs2-1*, *acs6-1*, and *acs2-1acs6-1*; overexpression lines *ACS2*-OE(#1), *ACS2*-OE(#3), *ACS6*-OE(#1), and *ACS6*-OE(#3); and complementation lines *ACS2*/*acs2-1*(#1), *ACS2*/*acs2-1*(#3), *ACS6*/*acs6-1*(#1), and *ACS6*/*acs6-1*(#3). *ACTIN8* was used as a reference gene. Experiments were repeated three times. Values are means ± SD (Student’s *t*-test; ^**^P < 0.01). **(B)** RT-qPCR analysis of *ACS6* mRNA level in *acs2-1* and *ACS2* mRNA level in *acs6-1*. Experiments were repeated three times with similar results. **(C)** Relative rate of water loss over time from detached rosette leaves of 28-day-old plants. All true leaves of five plants of the same line grown under identical conditions were collectively weighed every hour. The data represent the water loss percentage at a given time point, calculated as follows: [(initial weight – weight at each time point)/initial weight] × 100. Experiments were repeated at least three times with similar results. Values are means ± SD (Student’s *t*-test; *P < 0.05).

The expressions of *ACS2* and *ACS6* were modified in several genetic materials. For example, compared with WT, the loss-of-function mutant lines *acs2-1*, *acs6-1*, and *acs2-1acs6-1* showed significantly reduced *ACS2* or *ACS6* mRNA levels; the transgenic *ACS2*-OE(#1), *ACS2*-OE(#3), *ACS6*-OE(#1), and *ACS6*-OE(#3) over-expression lines had significantly elevated *ACS2* and *ACS6* mRNA levels, respectively; the transgenic complemented lines *ACS2*/*acs2-1*(#1), *ACS2*/*acs2-1*(#3), *ACS6*/*acs6-1*(#1), and *ACS6*/*acs6-1*(#3) exhibited no changes in *ACS2* or *ACS6* expression, respectively (Figure 1A). Interestingly, the expression levels of *ACS6* and *ACS2* were relatively unchanged in the single mutants *acs2-1* and *acs6-1*, respectively (Figure 1B). We first checked the growth phenotypes of the various ACS2/6 expression lines in response to drought caused by stopping watering for 12 days and the soil water content dropped to ∼39% (drought index refers to Yang et al., 2016). Compared with the control (normal watering), drought caused by stopping watering resulted in WT, *ACS2*/*acs2-1*(#1), *ACS6*/*acs6-1*(#1), *ACS2*-OE(#1), *ACS2*-OE(#3), *ACS6*-OE(#1), and *ACS6*-OE(#3) seedlings withered and even died as the drought intensifying, while the mutants *acs2-1*, *acs6-1*, and *acs2-1acs6-1* seedlings alleviated these wilting or drying symptoms (Supplementary Figure 1). Apparently ACS2/6 activation promoted dehydration and wilting of seedlings in response to drought treatment.

The rate of water evaporation from detached leaves of these lines was monitored. The water loss rate was decreased in *acs2-1*, *acs6-1*, and *acs2-1acs6-1* leaves, compared with that in WT (Figure 1C). Conversely, the water evaporation rate was significantly increased in *ACS2*-OE(#1) or *ACS6*-OE(#1) compared with that of WT, whereas no significant change was observed in *ACS2/acs2-1*(#1) or *ACS6*/*acs6-1*(#1) (Figure 1C). The other transgenic lines, such as *ACS2*-OE(#3), and *ACS6*-OE(#3), *ACS2/acs2-1*(#3), and *ACS6*/*acs6-1*(#3), also have the same phenotype (Supplementary Figure 2). Data suggest that ACS2/6 activation was positively correlated with the rate of water evaporation from detached leaves.

**FIGURE 2:**
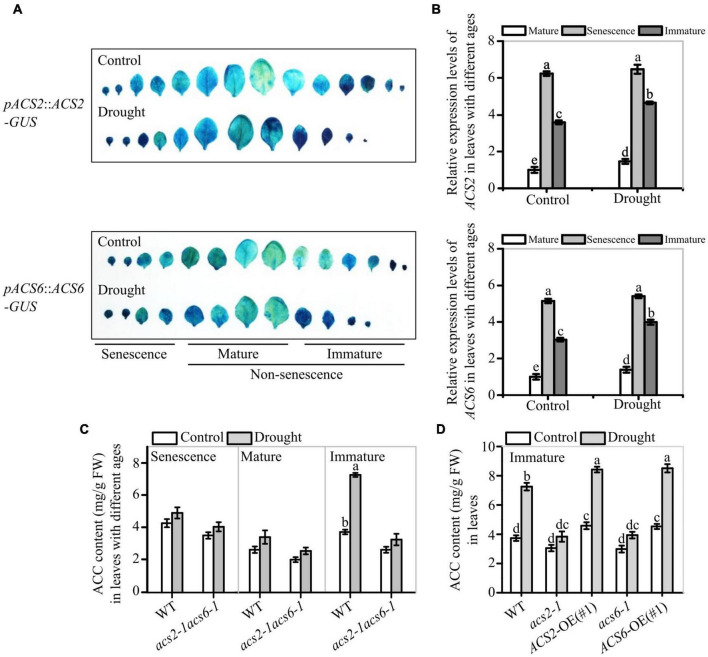
Effects of drought on ACS2 and ACS6 expressions and ACC accumulation in leaves of different ages. **(A)** ACS2 or ACS6 expression was monitored in immature, mature and senescence leaves of recombinant *pACS2*::*ACS2-GUS* or *pACS6*::*ACS6-GUS* lines, respectively, with or without stopping watering (drought) treatment. Representative images are shown. Experiments were repeated three times with similar results. **(B)** *ACS2* or *ACS6* expression was monitored by RT-qPCR in immature, mature and senescence leaves of WT, respectively, with or without drought treatment. Experiments were repeated three times with similar results. Values are means ± SD. Letters indicate significant differences (*P* < 0.05, two-way ANOVA). **(C,D)** HPLC analysis of ACC accumulation in leaves with different ages of 21-day-old seedlings with or without drought treatment. Experiments were repeated three times with similar results. Values are means ± SD. Letters indicate significant differences (*P* < 0.05, two-way ANOVA).

The characteristics of ACS2/6 expression in leaves were examined in response to drought treatment. Histochemical staining revealed that the higher GUS-marked ACS2/6 expression was in immature leaves, followed by senescent leaves, and then mature leaves in WT plants; After withholding water for 6 days, ACS2 and ACS6 expressions in WT were significantly increased in immature leaves, slightly increased in mature leaves, and unchanged in senescent leaves (Figure 2A). Meantime, quantitative real-time PCR showed the same results (Figure 2B). This is, ACS2 and ACS6 expressions were always higher in senescent leaves regardless of drought, whereas both expressions increased in response to drought in non-senescent leaves.

Next, the effects of ACS2/6 expression activity on ACC accumulation was analyzed in leaves. Under normal conditions, ACC mainly accumulated in immature and senescent leaves of WT seedlings, whereas ACC accumulated primarily in immature leaves in response to a 6-day halt in watering. More specifically, ACC levels were, respectively, 1.94- and 1.33-times higher in immature and mature leaves of WT after withholding water (Figure 2C). In contrast to WT, the double mutant *acs2-1acs6-1* did not significantly accumulate ACC in the immature leaves in response to drought (Figure 2C). In the meantime, ACC accumulation in immature leaves was more in *ACS2*-OE(#1) and *ACS6*-OE(#1) than that in WT and mutants (*acs2-1* or *acs6-1*) under drought stress (Figure 2D). These data suggest that drought-induced ACS2/6 expression and ACC accumulation was involved in the regulation of stomatal development and pattern in non-senescent leaves.

### ACS2/6 Activation Affected Stomatal Space on the Epidermis of Leaves Subjected to Drought Treatment

Evidences suggest that the ACS activity was required for stomatal development (Serna and Fenoll, 1996; Saibo et al., 2003; Young et al., 2004; Zhang et al., 2013; Lau et al., 2014; Yin et al., 2019), we thus analyzed the effects of ACS2/6 expression activity on stomatal density and stomatal clustering ratio on the leaf abaxial epidermis of *Arabidopsis* seedlings.

**FIGURE 3:**
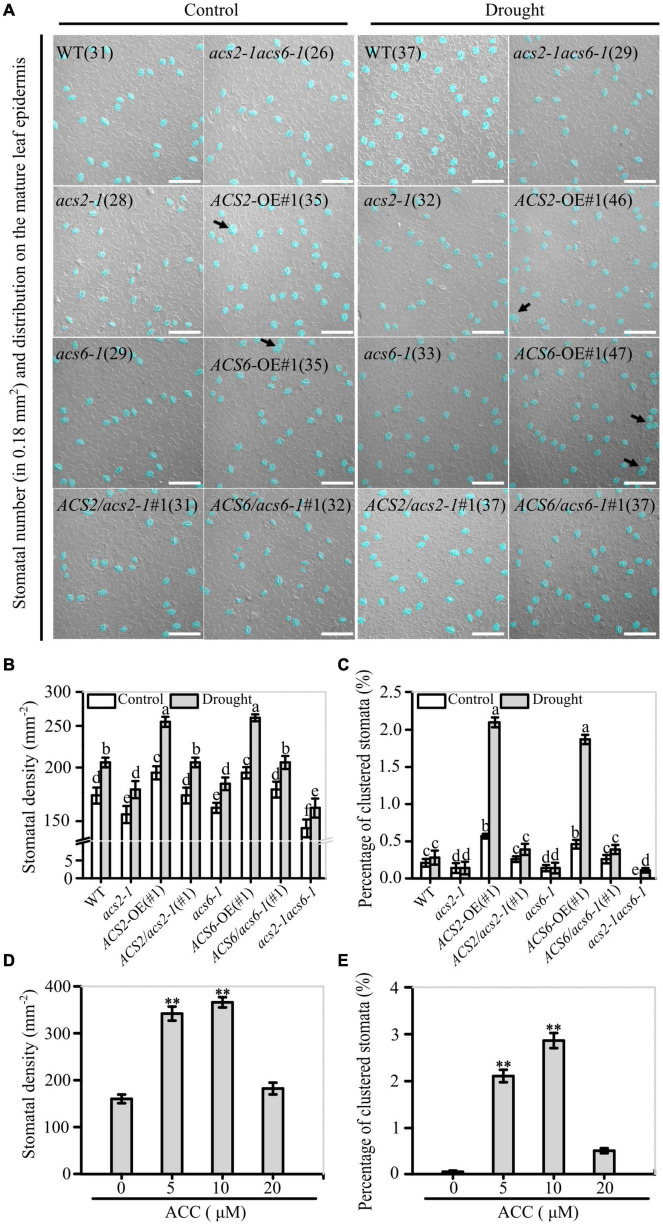
Correlation between ACS2/6 activation and stomatal density and rate of stomatal clustering. **(A)** Images of stomata distributed on strips of leaf abaxial epidermis under differential interference contrast (DIC) microscopy. Stomata and stomatal clusters are colored in blue for easier identification, and stomatal clusters are indicated by black arrows. The number of stomata is given in parentheses in each image. Sixth rosette were collected under drought or normal watering conditions from 28-day-old seedlings of the WT control; mutants *acs2-1*, *acs6-1*, and *acs2-1acs6-1*, overexpression lines *ACS2*-OE(#1) and *ACS6*-OE(#1), and complementation lines *ACS2*/*acs2-1*(#1) and *ACS6*/*acs6-1*(#1). Experiments were performed three times with similar results. The white scale bar represents 100 μm. **(B,C)** Statistical analysis of stomatal density **(B)** and percentage of clustered stomata **(C)** under normal and drought condition. Number of stomata and stomatal clusters on sixth leaves of 25 seedlings were counted. Values are means ± SD (*n* = 25 repeats). Letters indicate significant differences (*P* < 0.05, two-way ANOVA). **(D,E)** After treated immature leaves by ACC (0–20 μM) for 6 days, stomatal density **(D)** and percentage of clustered stomata **(E)** were analyzed, respectively. A total of 25 leaves from 25 seedlings were used to analyze the numbers of stomata and percentage of clustered stomata. Values are means ± SD. (Student’s *t*-test, ^**^P < 0.01).

Images of stomata and stomatal clusters on the abaxial epidermis of the sixth (count up from cotyledon) mature leaves of the various ACS2/6 expression lines under the control or drought conditions are shown in Figure 3A. Under normal watering conditions, there was no significant difference in the number of stomatal density, stomatal index and clustering rate between the mutants *acs2-1*, *acs6-1*, and *acs2-1acs6-1*, and over-expression lines *ACS2*-OE(#1), *ACS6*-OE(#1), and WT (Figure 3B), and also in other transgenic lines *ACS2*-OE(#3) and *ACS6*-OE(#3), *ACS2/acs2-1*(#3), and *ACS6*/*acs6-1*(#3) (Supplementary Figure 3A). After halting watering for 6 days, however, stomatal density was significantly reduced in *acs2-1* (177.8 ± 8.2 mm^–2^), *acs6-1* (183.8 ± 6.2 mm^–2^), and *acs2-1acs6-1* (161.3 ± 8.2 mm^–2^), but significantly increased in *ACS2*-OE(#1) (255.6 ± 6.9 mm^–2^) and *ACS6*-OE(#1) (261.1 ± 4.9 mm^–2^), compared with that in WT (205.6 ± 5.4 mm^–2^) (Figure 3B). Meantime, stomatal index on the leaf epidermis of WT was 25.3%, by comparison, the mutants *acs2-1* (19.9%), *acs6-1* (20.8%), and *acs2-1acs6-1* (20.2%) evidently reduced but *ACS2*-OE(#1) (28.5%) and *ACS6*-OE(#1) (28.5%) increased stomatal index (Supplementary Figure 3B). This is, the expression of ACS2/6 was a positive relationship with both stomatal density based on leaf area and stomatal index based on sum of epidermal cells and stomata. It is interesting to note that the percentage of the pairs of directly contacting stomata was significantly higher in *ACS2*-OE(#1) (2.1%) and *ACS6*-OE(#1) (1.87%), but was significantly lower in the mutants *acs2-1* (0.14%), *acs6-1* (0.14%) and *acs2-1acs6-1* (0.11%) than in WT (0.28%) (Figure 3C). In addition, the reduction of stomatal density did not occur in other mutants (*acs1-1*, *acs4-1*, *acs5-1*, *acs7-1*, *acs8-1*, *acs9-1*, and *acs11-1*) (Supplementary Figure 4). These data indicate that ACS2/6 activation reduced stomatal space, presented as an increase of stomatal density and the rate of clustered stomata on the leaf abaxial epidermis under drought treatment.

In addition, the validation experiments indicate that appropriate concentrations (5–10 μM) of ACC increased stomatal density and ratio of clustered stomata on the leaf epidermis of WT seedlings (Figures 3D,E). In contrast, ACS activity inhibitor AVG treatment significantly decreased stomatal density regardless of drought (Supplementary Figure 5). These observations suggest that ACS2/6 activation and ACC accumulation increased stomatal density and ratio of clustered stomata on the abaxial epidermis of leave, and these characteristic changes are consistent with the wilting or drying phenotype under drought conditions.

### SPEECHLESS Promoted the Expression of *ACS2* and *ACS6* Genes by Docking to Each of Their Promoter Region

The above data suggest that the promotion of ACS2/6 activation and ACC accumulation on stomatal density and clustered ratio on the leaf epidermis is similar to that of SPCH (Tripathi et al., 2016; Zoulias et al., 2018), we speculated that SPCH may mediate ACS2/6 expression activity. Although a profile list generated by genome-wide ChIP-based sequencing of the targets of SPCH included both *ACS2* and *ACS6* (Lau et al., 2014), direct experimental evidence was still lacking.

**FIGURE 4:**
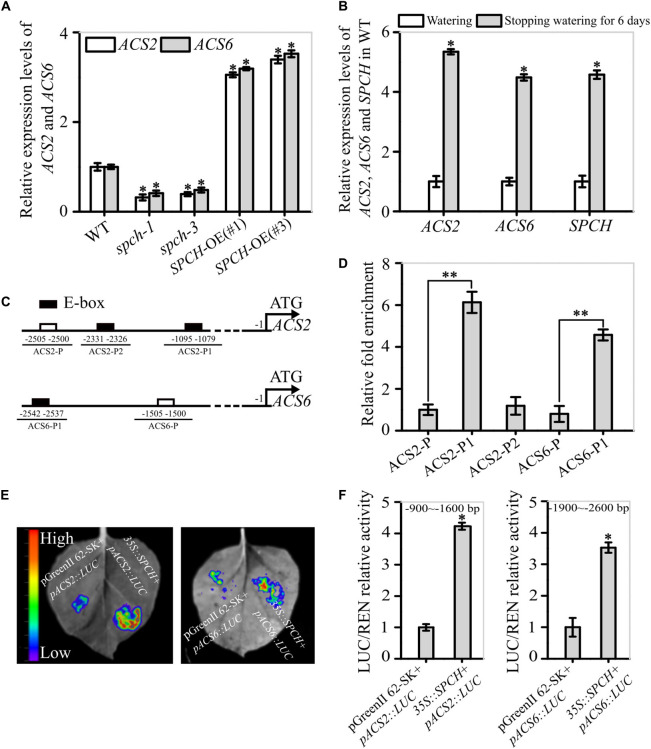
Evidence for the function of SPCH as a transcription factor of *ACS2* and *ACS6* genes. **(A)** Levels of *ACS2* and *ACS6* mRNA in immature leaves of WT control, loss-of-function *spch-1* and *spch-3* mutant, and *SPCH*-overexpressing plants of *SPCH*-OE(#1) and *SPCH*-OE(#3) based on RT-qPCR. Experiments were repeated three times with similar results. Values are means ± SD (Student’s *t*-test; *P < 0.05). **(B)** Relative levels of *ACS2*, *ACS6*, and *SPCH* mRNA transcripts in leaves of 21-day-old WT seedlings under normal watering conditions or after 6 days without watering. Experiments were repeated three times with consistent results. Values are means ± SD (Student’s *t*-test; *P < 0.05). **(C)** Diagram of the relative position of E-boxes (CACGTG or CGCGTG) and a reference DNA region in *ACS2* and *ACS6* gene promoters. Black rectangles indicate E-boxes in *ACS2* (ACS2-P1 and ACS2-P2) and *ACS6* (ACS6-P1) promoter regions, while white rectangles represent reference regions, namely randomly selected DNA fragments from *ACS2* (ACS2-P) and *ACS6* (ACS6-P) promoter regions. **(D)** Relative abundance of SPCH-immunoprecipitated DNA fragments under drought stress as determined by RT-qPCR. All experiments, which included three biological replicates, gave similar results. Values are means ± SD (Student’s *t*-test; ^**^P < 0.01). **(E,F)** Binding of SPCH protein to *ACS2* and *ACS6* genes in tobacco leaves in a transient transcription dual-luciferase assay. The size and intensity of LUC fluorescence signals recorded by IndiGO software are proportional to binding ability **(E)**. Relative binding ability was evaluated quantitatively by calculating the ratio of the fluorescence intensity of firefly luciferase (LUC) to that of an internal control, Renilla luciferase (REN) **(F)**. Values are means ± SD (*n* = 3). Asterisks indicate significant differences (*P < 0.05) compared with leaf regions injected with *Agrobacterium* harboring an empty vector.

To explore whether SPCH affect *ACS2* and *ACS6* expression, we checked mRNA levels of *ACS2* and *ACS6* in the *spch-1* and *spch-3* mutant seedlings, respectively. In order to explore the stomatal development on the epidermis of true leaves, the heterozygote of *spch-1* and *spch-3* were used, because the two homozygotes cannot grow true leaf (MacAlister et al., 2007; Pillitteri et al., 2007; Han and Torii, 2016). Observations indicated that both *spch-1* and *spch-3* had significantly reduced *ACS2* and *ACS6* mRNA levels, respectively, whereas *SPCH*-OE lines had significantly increased mRNA levels, compared with the WT control (Figure 4A). Interestingly, drought similarly induced the expressions of *SPCH*, *ACS2*, and *ACS6* genes (Figure 4B). Data implies that SPCH activity was positively correlated with the expression of *ACS2* and *ACS6*.

To confirm this experimentally, we used ChIP assays to detect the interaction between the transcription factor SPCH and the promoters of *ACS2* and *ACS6* under drought stress. The *in silico* analyses revealed three E-box motifs in the 3.0-kb promoter region of the *ACS2* gene: CGCGTG and CACGTG (at −1079 and −1090), collectively named ACS2-P1 because of their close proximity, and CACGTG (at −2326), designated as ACS2-P2. Only one E-box motif was present in the 3.0-kb promoter region of the *ACS6* gene: CACGTG (at −2537), named ACS6-P1 (Figure 4C). After randomly selecting DNA fragments from their promoter regions with the same length as the E-boxes (named ACS2-P and ACS6-P) as the reference, ChIP assays were performed to measure levels of immunoprecipitated DNA fragments by SPCH protein *in vivo*. In these assays, the abundance of DNA fragments from *ACS2* promoters ACS2-P1 and ACS2-P2 was, respectively, 6.13- and 1.18-fold higher than that of the control ACS2-P (Figure 4D). Similarly, the abundance of ACS6-P1 immunoprecipitated by SPCH protein was 4.57-fold higher than that of the control ACS6-P (Figure 4D). Next, we conducted transient transcription activity assays to verify the binding of SPCH to the promoters of *ACS2* and *ACS6*. According to the results, the fluorescence intensity of LUC linked to the specific promoter fragment of *ACS2* (−900 to −1600 bp, containing ACS2-P1) was increased in the presence of SPCH, with LUC activity 4.2-times higher than that of the blank LUC control (Figures 4E,F). Similarly, the activity of LUC linked to the promoter fragment of *ACS6* (−1900 to −2600 bp, containing ACS6-P1) in the presence of SPCH was 3.7-times higher than that of the blank LUC control (Figures 4E,F). This stimulatory effect was specific, as SPCH did not induce LUC activity alone or linked to the other promoter fragments of *ACS2* or *ACS6* gene (Supplementary Figure 6). This is, SPCH directed the transcription of *ACS2* or *ACS6* by docking to each of their promoter regions.

**FIGURE 5:**
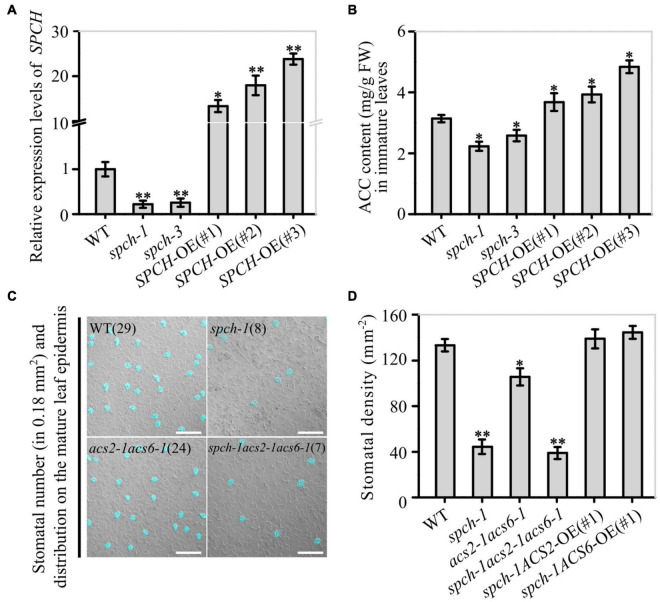
Correlation between SPCH activity and ACS2/6-dependent ACC accumulation and stomatal density and pattern. **(A,B)** RT-qPCR-based relative levels of *SPCH* mRNA transcripts and ACC content in immature leaves of WT control, loss-of-function mutant *spch-1* and *spch-3*, and *SPCH*-overexpressing *SPCH*-OE(#1), *SPCH*-OE(#2), and *SPCH*-OE(#3) plants. The *ACTIN8* gene was used as an internal control. The experiment was repeated three times with consistent results. Values are means ± SD (Student’s *t*-test; *P < 0.05; ^**^P < 0.01). **(C,D)** DIC images and statistical summary of stomatal density and patterning on the abaxial epidermis of *spch-1*, *acs2-1acs6-1*, and *spch-1acs2-1acs6-1* plants. Numbers of stomata are indicated in parentheses in each image. Stomata are false colored in blue for easier identification. The white scale bar represents 100 μm **(C)**. Stomata on sixth leaves of 25 seedlings were counted **(D)**. Values are means ± SD. Significant differences are indicated by asterisks (Student’s *t*-test; *P < 0.05; ^**^P < 0.01).

To verify that SPCH promoted *ACS2/6* expression, we monitored the effects of SPCH activity on ACC levels. The results showed that the SPCH-overexpressing lines *SPCH*-OE(#1), *SPCH*-OE(#2), and *SPCH*-OE(#3) had significantly increased ACC levels, but the mutants *spch-1* or *spch-3* had reduced ACC levels in immature leaves, as compared with the ACC levels in leaves of WT (Figures 5A,B). Evidently, SPCH-directed *ACS2/6* expression was directly related to ACC accumulation in immature leaves. Further observations helped to explain how SPCH mediated stomatal development *via ACS2/6-*dependent ACC production. The single mutant *spch-1*, the double mutant *acs2-1acs6-1*, and the triple mutant *spch-1acs2-1acs6-1* had significantly reduced stomatal densities, compared with WT (Figures 5C,D). In addition, the stomatal density on leaves was lower in the triple mutant *spch-1acs2-1acs6-1* than in its parents *spch-1* and *acs2-1acs6-1* (Figures 5C,D). Notably, ACS2- or ACS6-overexpression in *spch-1* reversed the reduction in stomatal density (Figure 5D). These observations suggest that SPCH activity induced ACS2/6 activation and ACC accumulation.

### ACS2/6-Generated 1-Aminocyclopropane-1-Carboxylate Accumulation Decreased the Expression Activity of STOMATAL DENSITY AND DISTRIBUTION 1 in Leaves

Evidences have shown that SDD1 expression reduces stomatal density and cluster (Yoo et al., 2010, 2019), but exogenously applied ACC increases stomatal density and cluster (Serna and Fenoll, 1996; Saibo et al., 2003; Acharya and Assmann, 2009). We therefore verified whether ACC cooperates with SDD1 to establish stomatal density and cluster.

**FIGURE 6:**
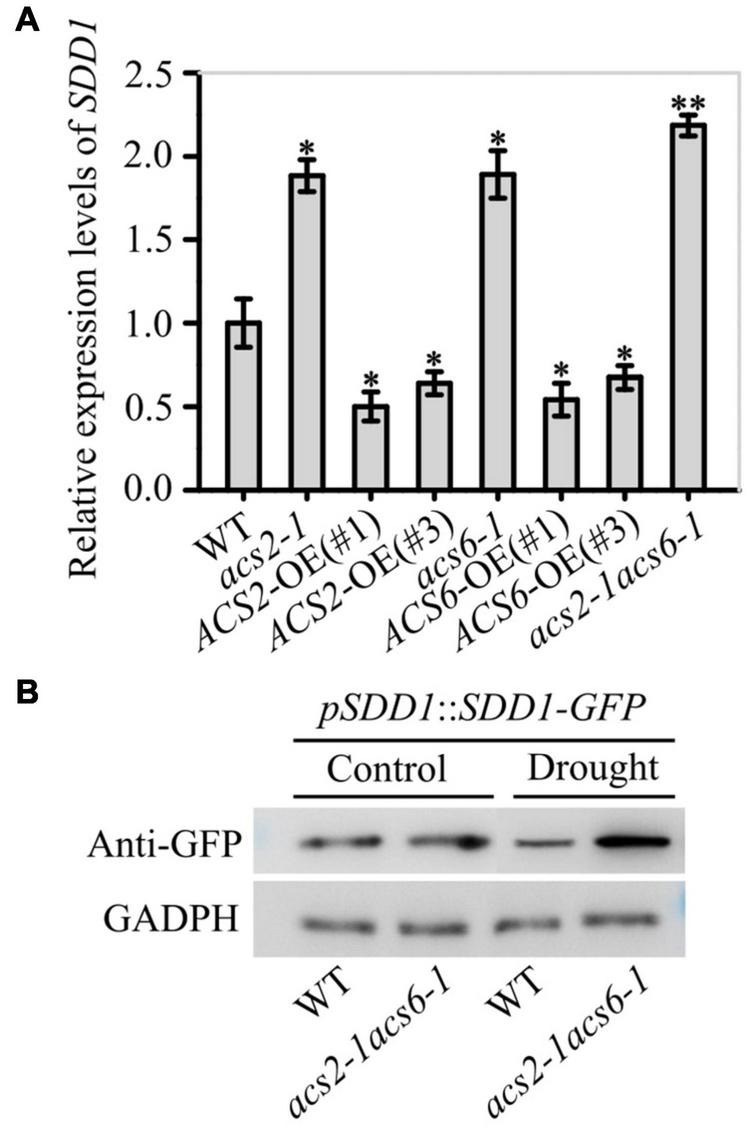
Inhibitory effects of ACS2/6 activation and ACC treatment on SDD1 expression and protein accumulation. **(A)** RT-qPCR-based relative levels of *SDD1* mRNA transcripts in immature leaves of WT, *acs2-1*, *acs6-1*, *acs2-1acs6-1*, *ACS2*-OE(#1), *ACS2*-OE(#3), *ACS6*-OE(#1), and *ACS6*-OE(#3) seedlings. The experiment was repeated three times with consistent results. Values are means ± SD (Student’s *t*-test; *P < 0.05; ^**^P < 0.01). **(B)** Evaluation of SDD1 protein levels by western blotting. The fusion protein GFP-SDD1 was collected from immature leaves of *pSDD1*::*SDD1-GFP*-expressing WT or *acs2-1acs6-1* lines with or without drought treatment. Levels of GFP-SDD1 fusion protein were determined using GFP antibody. GAPDH protein was used as a loading control. The experiment was repeated three times with consistent results.

The effect of ACS2/6-dependent ACC accumulation on SDD1 expression was surveyed in immature leaves. The *SDD1* mRNA levels in *acs2-1*, *acs6-1*, *acs2-1acs6-1*, *ACS2*-OE(#1), *ACS2*-OE(#3), *ACS6*-OE(#1), and *ACS6*-OE(#3) were, respectively, 1.88-, 1.89-, 2.18-, 0.50-, 0.64-, 0.54-, and 0.67-fold that in WT (Figure 6A). This result suggests that *SDD1* expression was negatively correlated with ACS2/6 activation in immature leaves. Further monitoring of SDD1 protein levels by western blotting indicated that GFP-marked SDD1 protein levels in immature leaves were higher in *acs2-1acs6-1* than in WT under drought conditions (Figure 6B). This result is consistent with the expectation that ACC treatment would reduce *SDD1* mRNA transcript levels in immature leaves of WT (Supplementary Figure 7A). As expected, ACC treatment reduced protein levels of SDD1 in WT leaves compared with the control (Supplementary Figure 7B). These data suggest that ACS2/6-generated ACC impeded *SDD1* expression and SDD1 protein levels, thereby increasing stomatal density and cluster on the leaf epidermis.

### 1-Aminocyclopropane-1-Carboxylate Buffered Ca^2+^ Activity in Stomatal Lineage Cells

Studies have shown that the trihelix transcription factor GTL1 is the inhibitor of SDD1 expression, and that Ca^2+^-loaded calmodulin can release the binding of GTL1 in the promoter region of SDD1 gene in stomatal lineage (Yoo et al., 2010, 2019; Weng et al., 2012; Virdi et al., 2015). That is to say, the increase of Ca^2+^ levels plays important roles in the establishment of stomatal space based on the SDD1 activity. Thus, it is required to monitor whether and how ACC mediates Ca^2+^ levels in stomatal lineage cells on the leaf epidermis under drought conditions.

We preliminarily evaluated the Ca^2+^ levels in stomatal lineage cells on the immature leaf epidermis by using the Ca^2+^ fluorescence probe Fluo-4/AM. Under normal conditions, the Ca^2+^ levels in stomatal lineage cells were slightly higher in *acs2-1acs6-1* than in WT. However, after halting watering for 6 days, the Ca^2+^ levels in stomatal lineage cells were higher in *acs2-1acs6-1* than in WT (Supplementary Figure 8). This hints that the decreased ACC accumulation increased Ca^2+^ levels in stomatal lineage cells.

**FIGURE 7:**
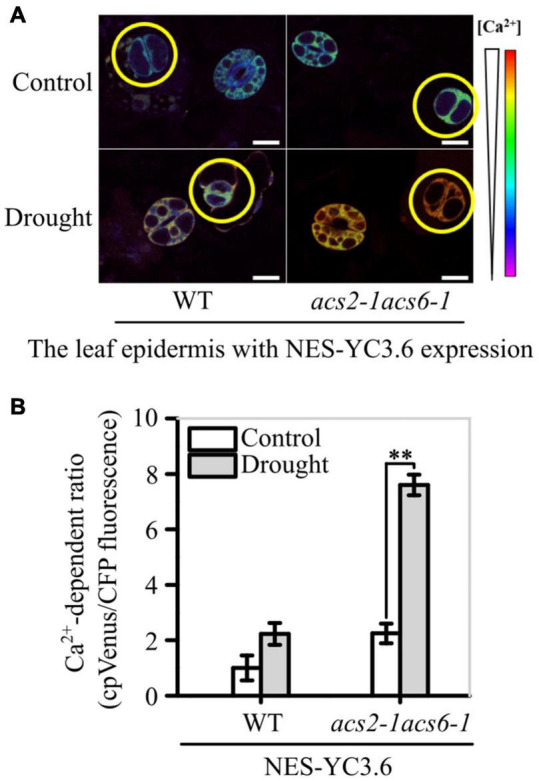
ACS2/6-generated ACC buffering of Ca^2+^ activity in stomatal lineage cells. **(A)** The relative fluorescence intensity of YC3.6 expression in stomatal lineage cells of immature leaves of NES-YC3.6-expressing WT or *acs2-1acs6-1* plants or by drought treatment. Representative images from at least 25 leaves in each line are shown. Pseudocolors in images correspond to relative Ca^2+^ levels according to the color scale on the right. Scale bar, 10 μm. **(B)** Evaluation of Ca^2+^ levels in stomatal lineage cells (labeled with yellow circle) of immature by calculating the ratio of the FRET acceptor cpVenus (at 525–555 nm) to the FRET donor CFP (at 465–499 nm). The experiment was repeated three times with consistent results. Values are means ± SD (Student’s *t*-test; ^**^P < 0.01).

The Ca^2+^-sensitive yellow cameleon protein YC3.6 has been developed as a Ca^2+^ biosensor (Krebs et al., 2012; Behera et al., 2017). After creating NES-YC3.6 expressing *acs2-1acs6-1* lines, both fluorescence-symbolized and cpVenus/CFP ratio-labeled Ca^2+^ levels were analyzed. Under normal conditions, the fluorescence intensity of YC3.6 protein was slightly higher in *acs2-1acs6-1* than in WT (Figure 7A). Moreover, the cpVenus/CFP ratio was slightly higher in *acs2-1acs6-1* than in WT (Figure 7B). After halting watering for 6 days, the fluorescence intensity of YC3.6 protein was significantly higher in *acs2-1acs6-1* than in WT (Figure 7A). Specifically, the Ca^2+^ levels in the stomatal lineage cells was 3.38-times higher in *acs2-1acs6-1* than in WT (Figure 7B). That is, ACC accumulation in leaves significantly reduced Ca^2+^ levels in stomatal lineage cells, which is the basis of decreasing SDD1 activity and of increasing stomatal density and clustered stomata.

## Discussion

**FIGURE 8:**
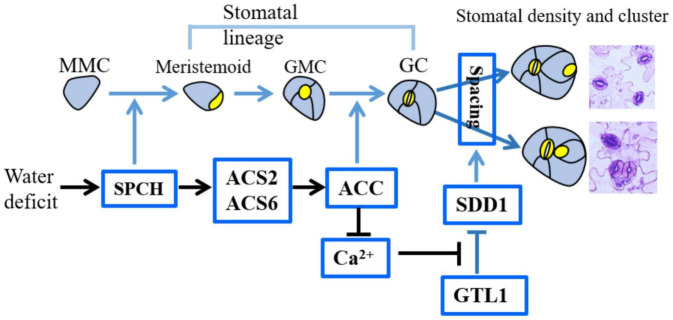
Diagram illustrating the role of ACS2/6 expression and ACC accumulation in the integration of SPCH-initiated stomatal development with SDD1-dependent stomatal spacing. In the top row depicting stomatal development, non-stomatal lineage cells, such as leaf epidermal cells, protodermal cells, and meristemoid mother cells (MMCs), are shown in blue–gray, while stomatal lineage cells, including meristemoid cells, guard mother cells (GMCs), and guard cell (GCs), are indicated in bright yellow. The factors involved in this study are shown in the bright blue box. Under water deficit conditions, SPCH increases *ACS2* and *ACS6* expressions. Next, ACS2/6-generated ACC accumulation may be involved in two processes: (1) promoting the transformation of GMCs into GCs, and (2) inducing a shortage of Ca^2+^ in stomatal lineage cells and decreasing SDD1 activity. As a consequence of these two processes, ACS2/6-generated ACC accumulation increases stomatal density, stomatal index and the rate of clustering. Relationships among these events are indicated by arrows: blue for conclusions drawn from the literature, and black for findings of the present study.

These findings first disclosed the mechanisms by which ACS2/6 activation directs the establishment of stomatal space by integrating SPCH signal with SDD1 function under drought conditions. A schematic overview of the inter-relationships among these processes is provided in Figure 8.

The activation of ACS2/6 lays the foundation for the ACC-dependent establishment of stomatal space in response to mild drought. According to our data, stomatal density (Figure 3B), stomatal index (Supplementary Figure 3B) and clustering ratio (Figure 3C) on the leaf epidermis were reduced in loss-of-function mutants *acs2-1*, *acs6-1*, and *acs2-1acs6-1*, but were increased in *ACS2-* and *ACS6-*overexpression lines; in other words, drought-activated ACS2/6 increased stomatal density, stomatal index and clustering, and these facilitated stomata-based water evaporation, in turn, seedlings withered and some even died with drought escalating. This finding provides a genetic explanation for the decrease in stomatal density and clustering caused by ACS activity inhibitor AVG in *Arabidopsis* (Serna and Fenoll, 1996; Saibo et al., 2003; Yin et al., 2019), and also provide theoretical explanations why ACS2- or ACS6-deficient rice (Zhang et al., 2013) or maize (Young et al., 2004) are less sensitive to water deficit than are WT controls. Considering the specificity of ACS activity (Tsuchisaka and Theologis, 2004; Tsuchisaka et al., 2009; Han et al., 2019; Lv et al., 2019), we presume that ACS2/6 activation is specific to stomatal development and spacing on the leaf epidermis when *Arabidopsis* seedlings are under drought. Because drought can induce ACS2/6 activation and ACC accumulation (Catalá et al., 2014; Dubois et al., 2018), the observed increase in stomatal density and cluster under drought conditions (Figures 2, 3) is easily understandable. Simply, ACS2/6-dependent ACC accumulation increases the susceptibility of seedlings to drought. Nevertheless, we could not rule out the possibility that ethylene followed ACC accumulation to be involved in stomatal density and cluster setting, because an ethylene perceiving blocker silver ions (Ag^2+^) mimicked AVG to establish stomatal density and cluster on the cotyledon epidermis of Arabidopsis (Serna and Fenoll, 1996). Furthermore, activated ACS2 and ACS6 may function in parallel, as growth phenotypes (Supplementary Figure 1), stomatal densities and clustering (Figure 3) were similar among *acs2-1*, *acs6-1*, and *acs2-1acs6-1* mutants, and the expressions of *ACS6* and *ACS2* were relatively unaffected in the two single mutants *acs2-1* and *acs6-1*, respectively (Figure 1). Importantly, our results show that SPCH separately regulates the expression of *ACS2* and *ACS6* by binding to their promoters (Figure 4). We speculate that ACS2 and ACS6 jointly ensure plants to fully respond to frequent drought stimuli.

ACS2/6-dependent ACC production is a turning point from SPCH-based stomatal development to the SDD1-directed establishment of stomatal space. Evidence for this conclusion is as follows: First, in line with a previous prediction (Lau et al., 2014), our results provide evidence that SPCH acts as a transcription factor to control the expression of *ACS2* and *ACS6* (Figure 4). The ability of SPCH to promote *ACS2* and *ACS6* expression was evidenced by the fact, for example, that *spch-1* and *spch-3* mutants showed reduced expressions of these genes (Figure 4A) and ACC content, whereas SPCH overexpression led to increased ACC levels (Figure 5B). This finding explains why plant tolerance to osmotic stress requires reduced SPCH activity (Han and Torii, 2016; Tripathi et al., 2016; Zoulias et al., 2018). Second, ACC mimics SPCH to reduce SDD1 activity, thereby increasing stomatal density and cluster (Figure 6 and Supplementary Figure 7). The mutants *acs2-1*, *acs6-1*, and *acs2-1acs6-1* seedlings (Figure 2) mimicked transgenic *SDD1-*overexpressing plants by showing reduced stomatal density and cluster on leaves. Consistent with this observation, both the *sdd1-1* mutant (Von Groll et al., 2002) and ACS2*-* and ACS6*-*overexpressing plants (Figure 3) exhibited increased stomatal density and cluster on the leaves. Third, ACC-associated Ca^2+^ insufficiency reduced SDD1 activity, or, alternatively, Ca^2+^ activity, in stomatal lineage cells, so that ACC levels were linked to SDD1 expression. Our findings indicated that Ca^2+^ levels in stomatal lineage cells on the leaf epidermis were higher in *acs2-1acs6-1* plants than in WT (Figure 7 and Supplementary Figure 8). This suggests that ACC accumulation inhibits SDD1 activity by controlling Ca^2+^ activity in stomatal lineage cells. This result is reasonable because a Ca^2+^ shortage can stabilize the binding of GTL1 to the *SDD1* promoter to prevent its expression in stomatal lineage cells (Yoo et al., 2019). These findings explicate the mechanisms in the recent discovery that Ca^2+^ activity intensifies stomata-based water evaporation from leaves of *Arabidopsis* seedlings under drought conditions (Teardo et al., 2019).

In summary, mild drought induces ACS2/6 activation and ACC accumulation, and thus reduces stomatal space, or increases stomatal density and cluster. This increase may be an adaptive response to mild drought, as the increase of stomata is beneficial to water absorption of plant roots, according to the view of evolution from aquatic to terrestrial (Croxdale, 2000; van Veen and Sasidharan, 2021). But this increase may also lead to wilting and drying of plants if drought intensifies and escalates. These findings provide a clear evidence that moderate drought increases stomata density and cluster, and a novel evidence that ACS2/6 activation is the key factor in the establishment of stomatal density and clustered ratio on the Arabidopsis leaf epidermis under moderate drought.

## Data Availability Statement

The datasets presented in this study can be found in online repositories. The names of the repository/repositories and accession number(s) can be found in the article/Supplementary Material.

## Author Contributions

JJ conceived the study and supervised the whole research. M-ZJ, L-YL, and CG performed experiments and analyzed the data. JJ and M-ZJ wrote the manuscript. All authors approved the submitted version.

## Conflict of Interest

The authors declare that the research was conducted in the absence of any commercial or financial relationships that could be construed as a potential conflict of interest.

## Publisher’s Note

All claims expressed in this article are solely those of the authors and do not necessarily represent those of their affiliated organizations, or those of the publisher, the editors and the reviewers. Any product that may be evaluated in this article, or claim that may be made by its manufacturer, is not guaranteed or endorsed by the publisher.

## Footnotes

1
http://signal.salk.edu
