# Supplementary material for: Activation of 1-Aminocyclopropane-1-Carboxylic Acid Synthases Sets Stomatal Density and Clustered Ratio on Leaf Epidermis of Arabidopsis in Response to Drought
Source: Front Plant Sci. 2021 Dec 6;12:758785. doi: 10.3389/fpls.2021.758785 (PMC8685546; doi:10.3389/fpls.2021.758785)
Supplement: Supplementary file 1 [file Table_1.DOCX]

**Table 1** Primer list.

| **Primers for screening of homozygous mutant plants** | | |
| --- | --- | --- |
| *acs2-1*-LP | | AAGGATCAAACCGTTGTTTCC |
| *acs2-1*-RP  LBa1 | | CAAGATCGTCGAGAAAGCATC  TGGTTCACGTAGTGGGCCATCG |
| *acs6-1*-LP | | CACTTGGTGAACAATCACACG |
| *acs6-1*-RP | | GCTTGCCTGAATTCAGACAAG |
| LB1 | | GCCTTTTCAGAAATGGATAAATAGCCTTGCTTCC |
| *spch-1*-LP | | ATGTTGAGACGAGAACAGA |
| *spch-1*-RP | | GATAATCTTTAAAACTCAC |
| *spch-3*-LP | | TTTCCCTTTGCAATATGCAAC |
| *spch-3*-RP | | GGACTTCGGCGTAGGTTTTAC |
| LB3 | | TTCATAACCAATCTCGATACAC |
| **Primers for cloning** | | |
| *ACS6*-OE-F | | TCCCCCGGGATGGTGGCTTTTGCAACAGAG |
| *ACS6*-OE-R | | GGGGTACCAGTCTGTGCACGGTAGCGGGAAGGC |
| *pACS6::ACS6-GUS*-F | | TGGCTGCAGGTCGACGGATCCATACATGAACAAAGATAAGTATTAGTAGT  ACACTAAT |
| *pACS6::ACS6-GUS*-R | | TCTTAGAATTCCCGGGGATCCTTTTTGTTTCTTCTTTAATATAGGTTTCTTT |
| *ACS6*/*acs6-1*-F | | GACCTGCAGGCATGCAAGCTTTTATTCATCGACCACCCACATG |
| *ACS6*/*acs6-1*-R | | ACGACGGCCAGTGCCAAGCTTAGTCTGTGCACGGACTAGCGG |
| *SPCH*-OE-F | | CGCTCTAGAATGCAGGAGATAATACCGGA |
| *SPCH*-OE-R | | CGGGGTACCGCAGAATGTTTGCTGAAT |
| *pSDD1::SDD1*-*GFP*-F | | GACCTGCAGGCATGCAAGCTTCGAGCAAATCTTGACCCACTTG |
| *pSDD1::SDD1*-*GFP*-R | | ACGACGGCCAGTGCCAAGCTTGTTAGTCTTCAAGGTTACAGAGATTGGA |
| **Primers for qRT-PCR** | | |
| *ACTIN8-*F | | GCAGACCGTATGAGCAAAGA |
| *ACTIN8-*R | | GAGGGAAGCAAGGATAGAAC |
| *ACS2-*F | | AGATCGTCGAGAAAGCATCTG |
| *ACS2-*R | | GAAGAGGTGAGTGTGGTGAC |
| *ACS6-*F | | CTGAATCTATTGTCTAAAATCGC |
| *ACS6-*R | | ACGCATCAAATCTCCACAAAG |
| *SPCH-*F | | CTCAAAACGGTGTCGCATAAGATCC |
| *SPCH-*R | | TGTTTGCTGAATTTGTTGAGCCAGT |
| *SDD1-*F | | GATTTCCCGCTGTGGTCAGA |
| *SDD1-*R | | TCCGCGATCACAAATCACCA |
| **Primers for ChIP** | | |
| *ACS2*-P-F | | ATATGCACGTCGGTGGAATG |
| *ACS2*-P-R | | TGAATGGGCTTATGGCCTGT |
| *ACS2*-P1-F | | GAGTTTTTGACATTCAGACG |
| *ACS2*-P1-R | | AGTTGGTGGGTTTGGACTCTTT |
| *ACS2*-P2-F | | ACTTTGACATGATCACTGTG |
| *ACS2*-P2-R | | CTTAACATAAATGCTGACTC |
| *ACS6*-P-F | | GAGACAGATGGAATTCTAC |
| *ACS6*-P-R | | AGATCTCCACCTTCTTTT |
| *ACS6*-P1-F | | CAATCTAATACTGTGTGGG |
| *ACS6*-P1-R | | GTTCTTGGGTTGAGAAACTT |
| **Primers for transient transcription dual-luciferase** | | |
| SPCH-SK-F | CGCTCTAGAACTAGTGGATCCATGCAGGAGATAATACCGGATTTT | |
| SPCH-SK-R | GATAAGCTTGATATCGAATTCGCAGAATGTTTGCTGAATTTGTTG | |
| ACS2-LUC  (-1~-1000)-F | TTCCTGCAGCCCGGGGGATCCTACAAAAAAAAATCAAGATATAAATAACTA  ACTC | |
| ACS2-LUC  (-1~-1000)-R | CGCTCTAGAACTAGTGGATCCTTGCTGTGTCAATTCTCACTTCTTT | |
| ACS2-LUC  (-900~-1600)-F | TTCCTGCAGCCCGGGGGATCCTACCTGCATTTCATGAGAAACACA | |
| ACS2-LUC  (-900~-1600)-R | CGCTCTAGAACTAGTGGATCCTAACCACAACAATTGTTAGTAGATCATATATG | |
| ACS2-LUC  (-1500~-2400)-F | TTCCTGCAGCCCGGGGGATCCCGTGAATCTTCTTCTCTTAAAAACAAA | |
| ACS2-LUC  (-1500~-2400)-R | CGCTCTAGAACTAGTGGATCCGAAACATTGTAGCTTTGTGAATATATTATTT | |
| ACS6-LUC  (-1~-1000)-F | TTCCTGCAGCCCGGGGGATCCAGAAAATTGTTATTAAAATTTTCTTAGCAA | |
| ACS6-LUC  (-1~-1000)-R | CGCTCTAGAACTAGTGGATCCTTTTTGTTTCTTCTTTAATATAGGTTTCTTT | |
| ACS6-LUC  (-900~-2000)-F | TTCCTGCAGCCCGGGGGATCCAACATCAGTCTGATAAAAAAAAAGTTCTT | |
| ACS6-LUC  (-900~-2000)-R | CGCTCTAGAACTAGTGGATCCCATGCGATTGTAATAACTTATACATAAAATT | |
| ACS6-LUC  (-1900~-2600)-F | TTCCTGCAGCCCGGGGGATCCTAAATTATTTATTTAAATTTTAAATTATAAAA  AAAGA | |
| ACS6-LUC  (-1900~-2600)-R | CGCTCTAGAACTAGTGGATCCATCTTTTTTCTGTATTTTTATTTGTTGCT | |
