# Supplementary material for: Activation of 1-Aminocyclopropane-1-Carboxylic Acid Synthases Sets Stomatal Density and Clustered Ratio on Leaf Epidermis of Arabidopsis in Response to Drought
Source: Front Plant Sci. 2021 Dec 6;12:758785. doi: 10.3389/fpls.2021.758785 (PMC8685546; doi:10.3389/fpls.2021.758785)
Supplement: Supplementary file 2 [file Presentation_1.PPTX]

## Slide 1
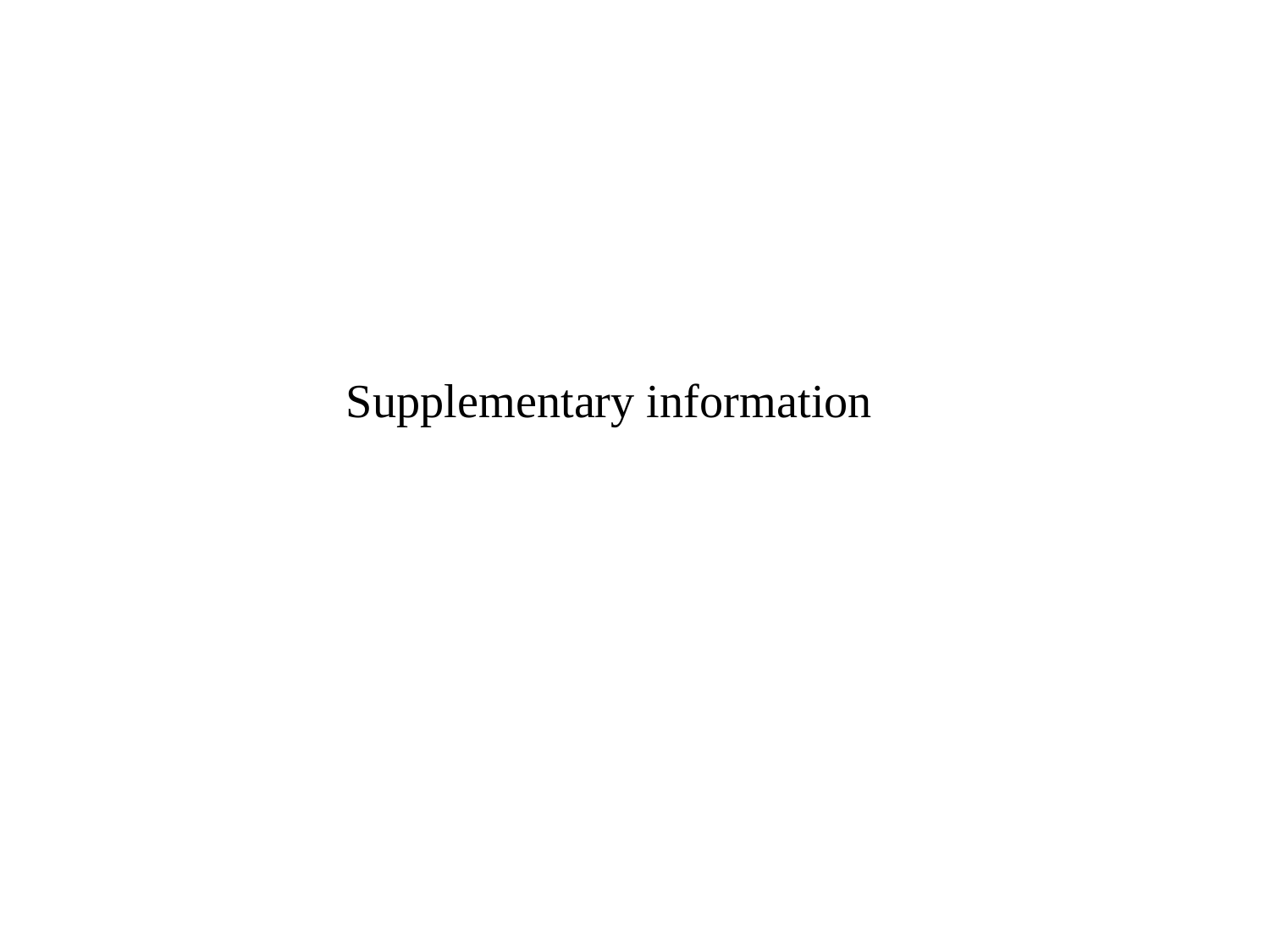

Supplementary information

## Slide 2
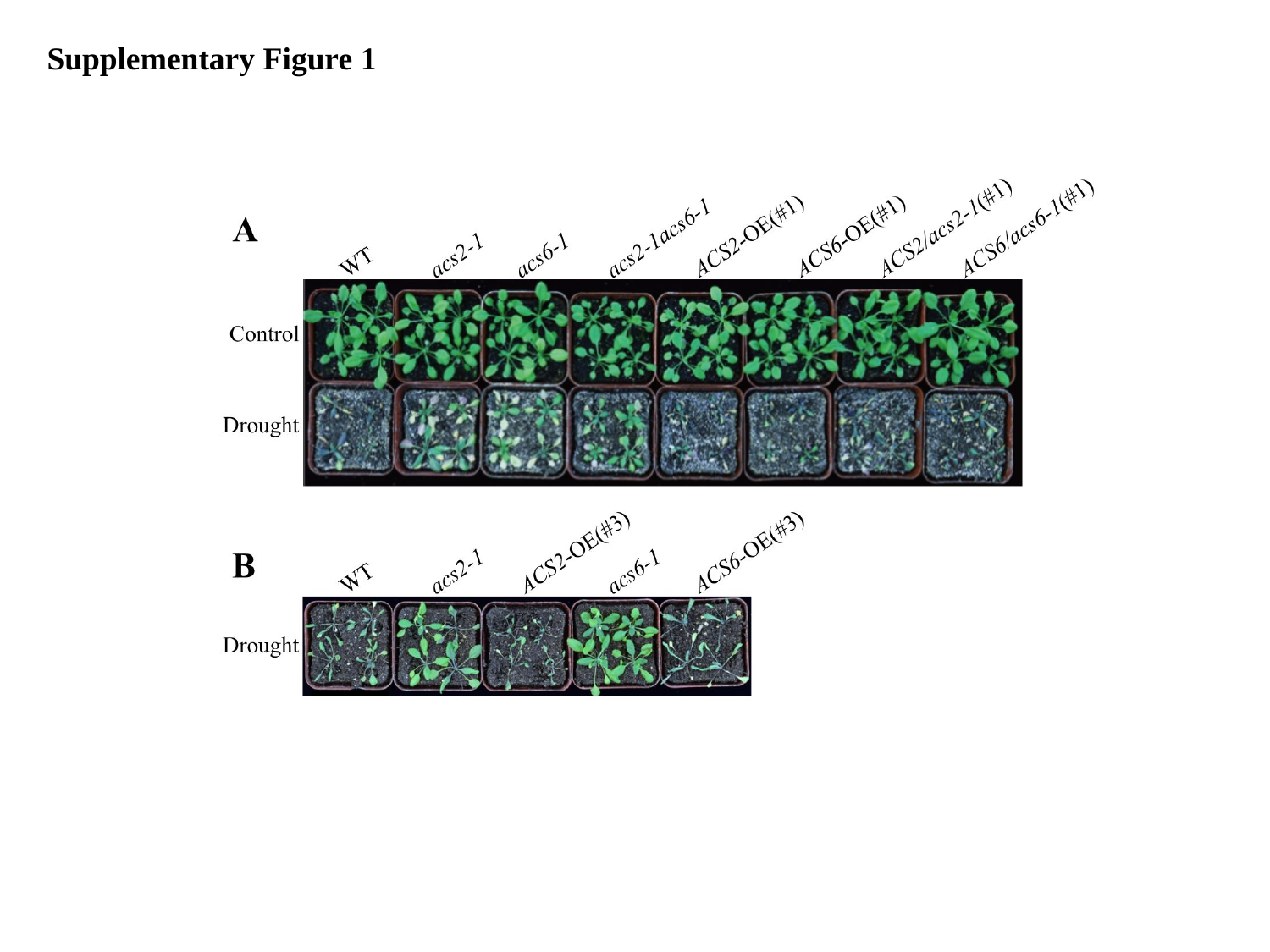

Supplementary Figure 1

## Slide 3
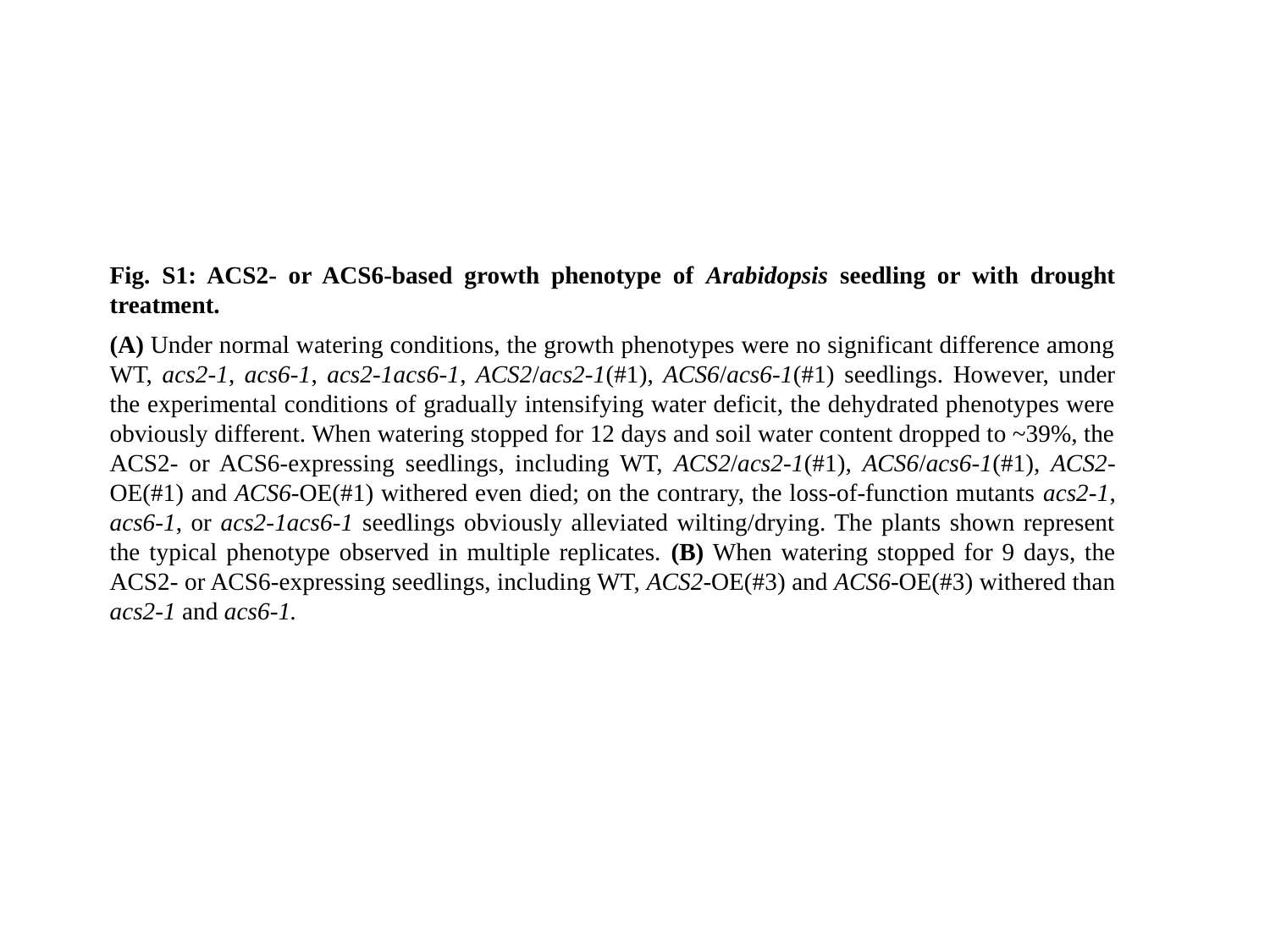

Fig. S1: ACS2- or ACS6-based growth phenotype of Arabidopsis seedling or with drought treatment.
(A) Under normal watering conditions, the growth phenotypes were no significant difference among WT, acs2-1, acs6-1, acs2-1acs6-1, ACS2/acs2-1(#1), ACS6/acs6-1(#1) seedlings. However, under the experimental conditions of gradually intensifying water deficit, the dehydrated phenotypes were obviously different. When watering stopped for 12 days and soil water content dropped to ~39%, the ACS2- or ACS6-expressing seedlings, including WT, ACS2/acs2-1(#1), ACS6/acs6-1(#1), ACS2-OE(#1) and ACS6-OE(#1) withered even died; on the contrary, the loss-of-function mutants acs2-1, acs6-1, or acs2-1acs6-1 seedlings obviously alleviated wilting/drying. The plants shown represent the typical phenotype observed in multiple replicates. (B) When watering stopped for 9 days, the ACS2- or ACS6-expressing seedlings, including WT, ACS2-OE(#3) and ACS6-OE(#3) withered than acs2-1 and acs6-1.

## Slide 4
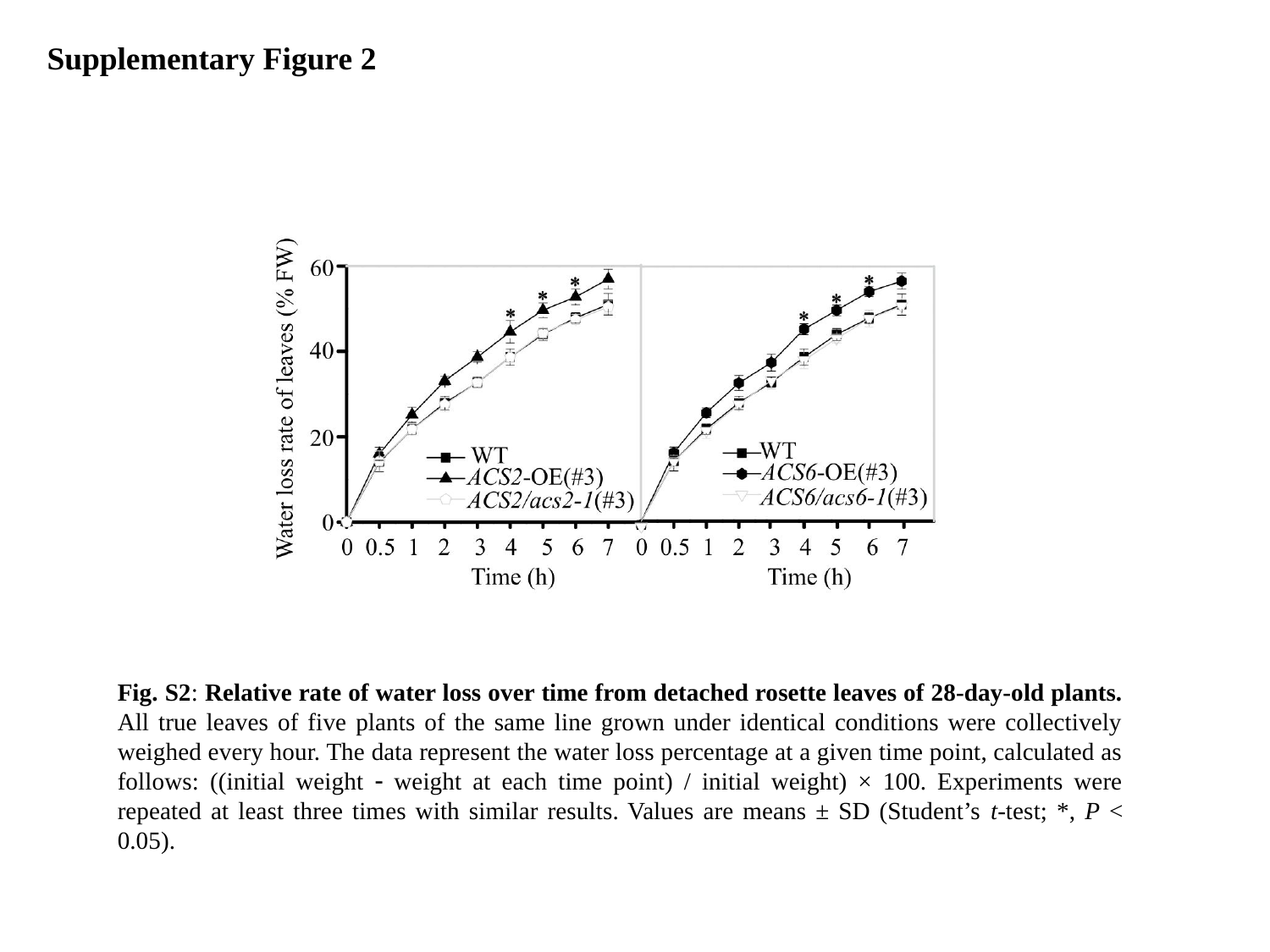

Supplementary Figure 2
Fig. S2: Relative rate of water loss over time from detached rosette leaves of 28-day-old plants. All true leaves of five plants of the same line grown under identical conditions were collectively weighed every hour. The data represent the water loss percentage at a given time point, calculated as follows: ((initial weight − weight at each time point) / initial weight) × 100. Experiments were repeated at least three times with similar results. Values are means ± SD (Student’s t-test; *, P < 0.05).

## Slide 5
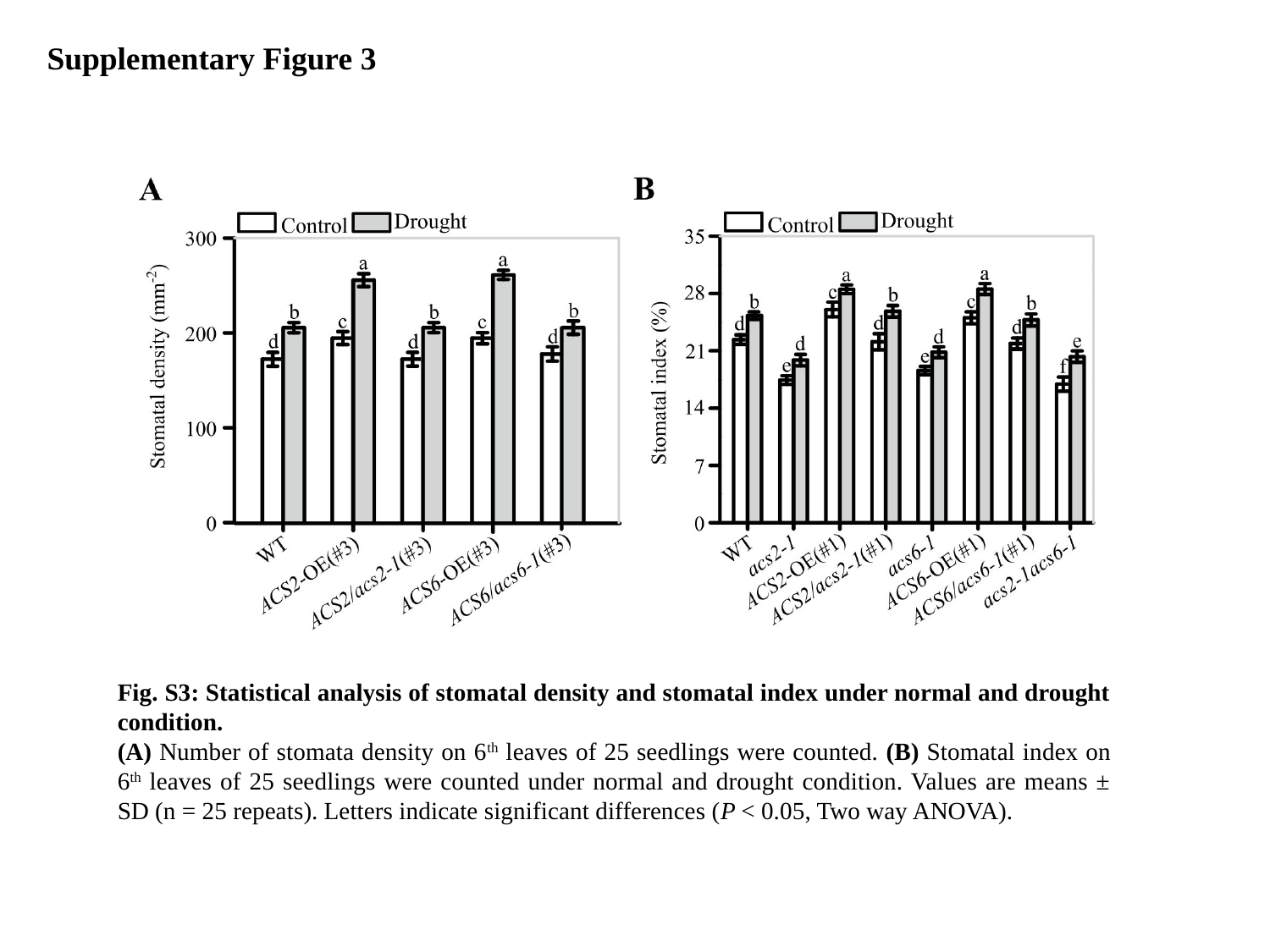

Supplementary Figure 3
Fig. S3: Statistical analysis of stomatal density and stomatal index under normal and drought condition.
(A) Number of stomata density on 6th leaves of 25 seedlings were counted. (B) Stomatal index on 6th leaves of 25 seedlings were counted under normal and drought condition. Values are means ± SD (n = 25 repeats). Letters indicate significant differences (P < 0.05, Two way ANOVA).

## Slide 6
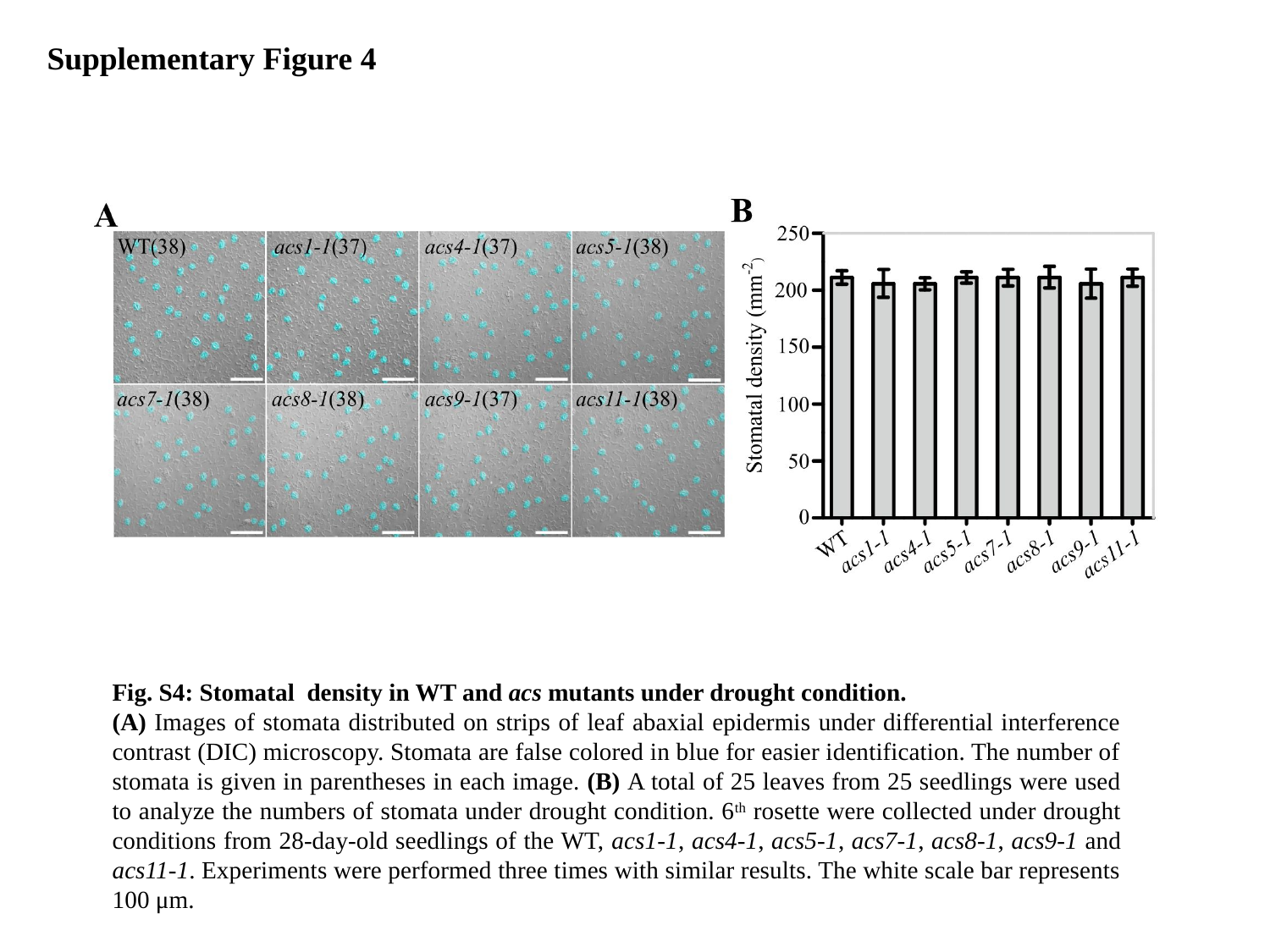

Supplementary Figure 4
Fig. S4: Stomatal density in WT and acs mutants under drought condition.
(A) Images of stomata distributed on strips of leaf abaxial epidermis under differential interference contrast (DIC) microscopy. Stomata are false colored in blue for easier identification. The number of stomata is given in parentheses in each image. (B) A total of 25 leaves from 25 seedlings were used to analyze the numbers of stomata under drought condition. 6th rosette were collected under drought conditions from 28-day-old seedlings of the WT, acs1-1, acs4-1, acs5-1, acs7-1, acs8-1, acs9-1 and acs11-1. Experiments were performed three times with similar results. The white scale bar represents 100 μm.

## Slide 7
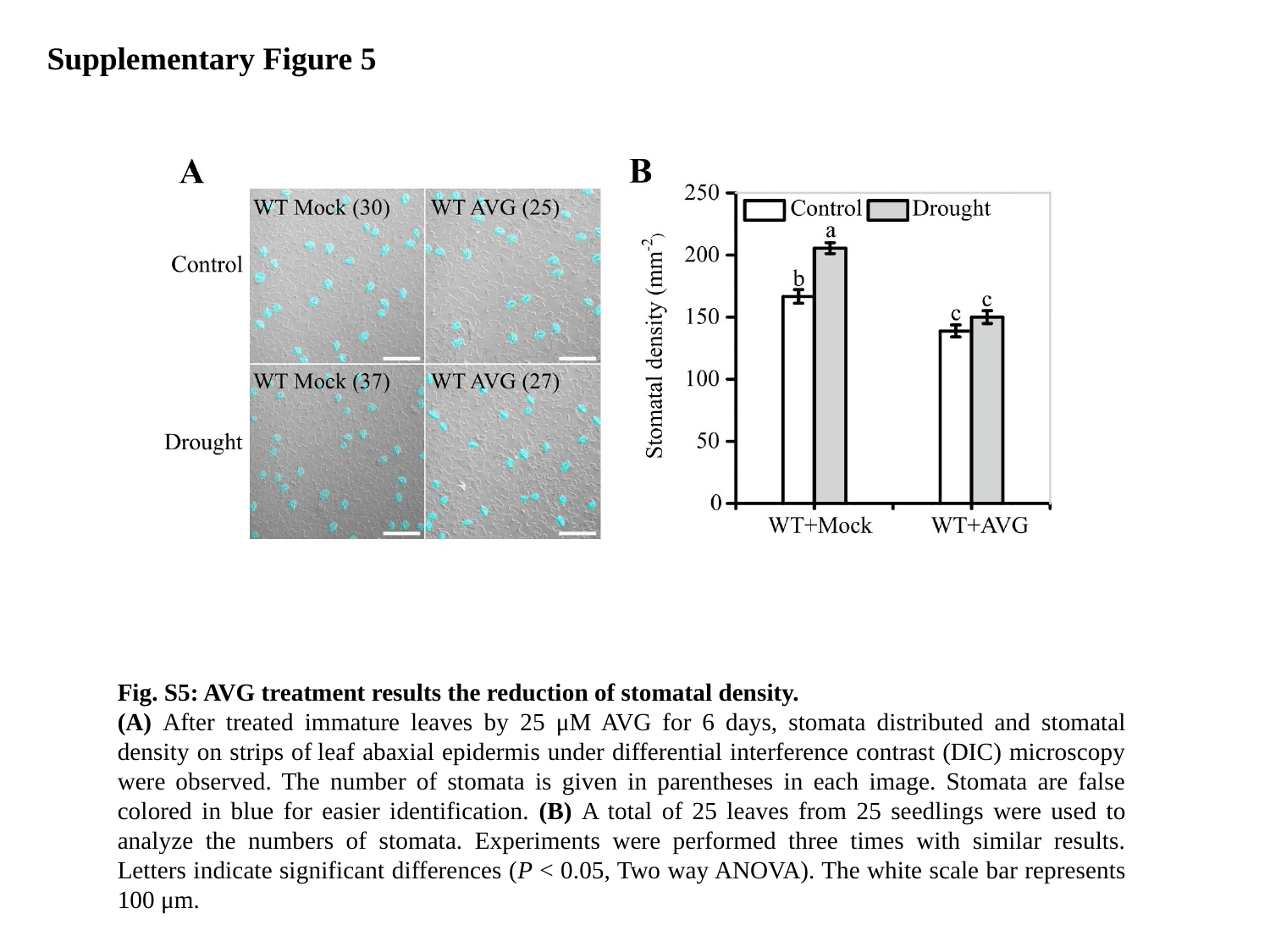

Supplementary Figure 5
Fig. S5: AVG treatment results the reduction of stomatal density.
(A) After treated immature leaves by 25 μM AVG for 6 days, stomata distributed and stomatal density on strips of leaf abaxial epidermis under differential interference contrast (DIC) microscopy were observed. The number of stomata is given in parentheses in each image. Stomata are false colored in blue for easier identification. (B) A total of 25 leaves from 25 seedlings were used to analyze the numbers of stomata. Experiments were performed three times with similar results. Letters indicate significant differences (P < 0.05, Two way ANOVA). The white scale bar represents 100 μm.

## Slide 8
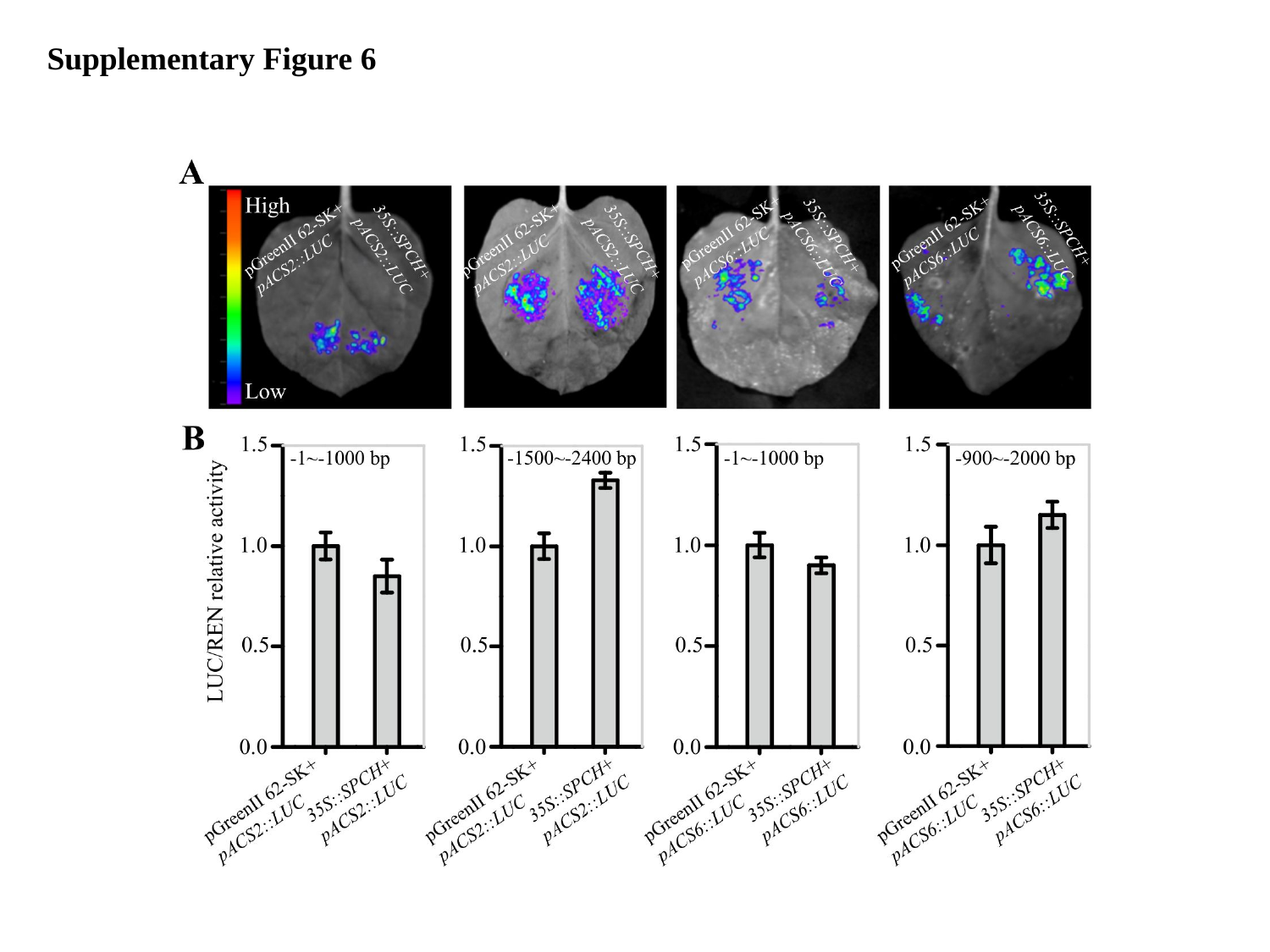

Supplementary Figure 6

## Slide 9
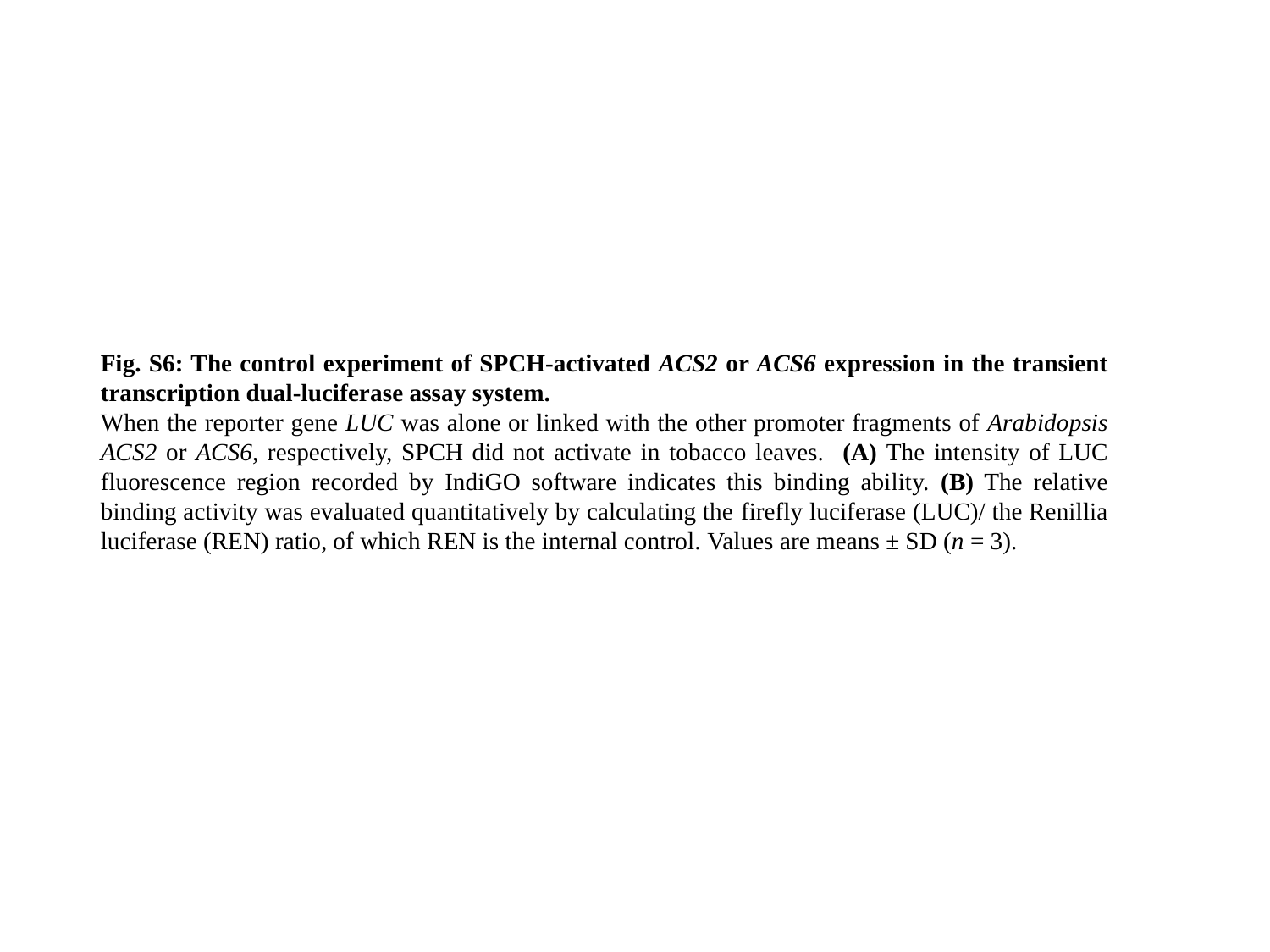

Fig. S6: The control experiment of SPCH-activated ACS2 or ACS6 expression in the transient transcription dual-luciferase assay system.
When the reporter gene LUC was alone or linked with the other promoter fragments of Arabidopsis ACS2 or ACS6, respectively, SPCH did not activate in tobacco leaves. (A) The intensity of LUC fluorescence region recorded by IndiGO software indicates this binding ability. (B) The relative binding activity was evaluated quantitatively by calculating the firefly luciferase (LUC)/ the Renillia luciferase (REN) ratio, of which REN is the internal control. Values are means ± SD (n = 3).

## Slide 10
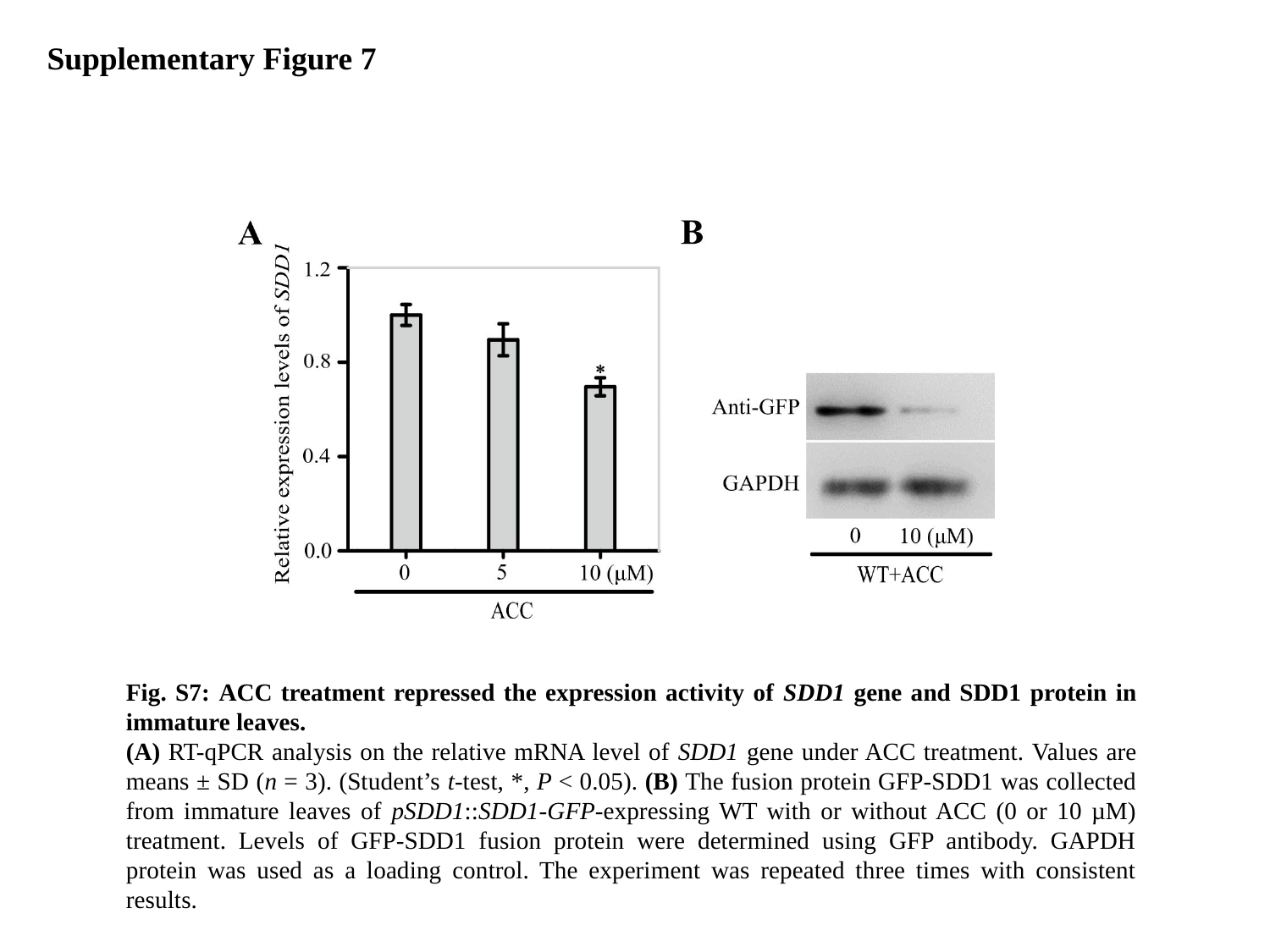

Supplementary Figure 7
Fig. S7: ACC treatment repressed the expression activity of SDD1 gene and SDD1 protein in immature leaves.
(A) RT-qPCR analysis on the relative mRNA level of SDD1 gene under ACC treatment. Values are means ± SD (n = 3). (Student’s t-test, *, P < 0.05). (B) The fusion protein GFP-SDD1 was collected from immature leaves of pSDD1::SDD1-GFP-expressing WT with or without ACC (0 or 10 µM) treatment. Levels of GFP-SDD1 fusion protein were determined using GFP antibody. GAPDH protein was used as a loading control. The experiment was repeated three times with consistent results.

## Slide 11
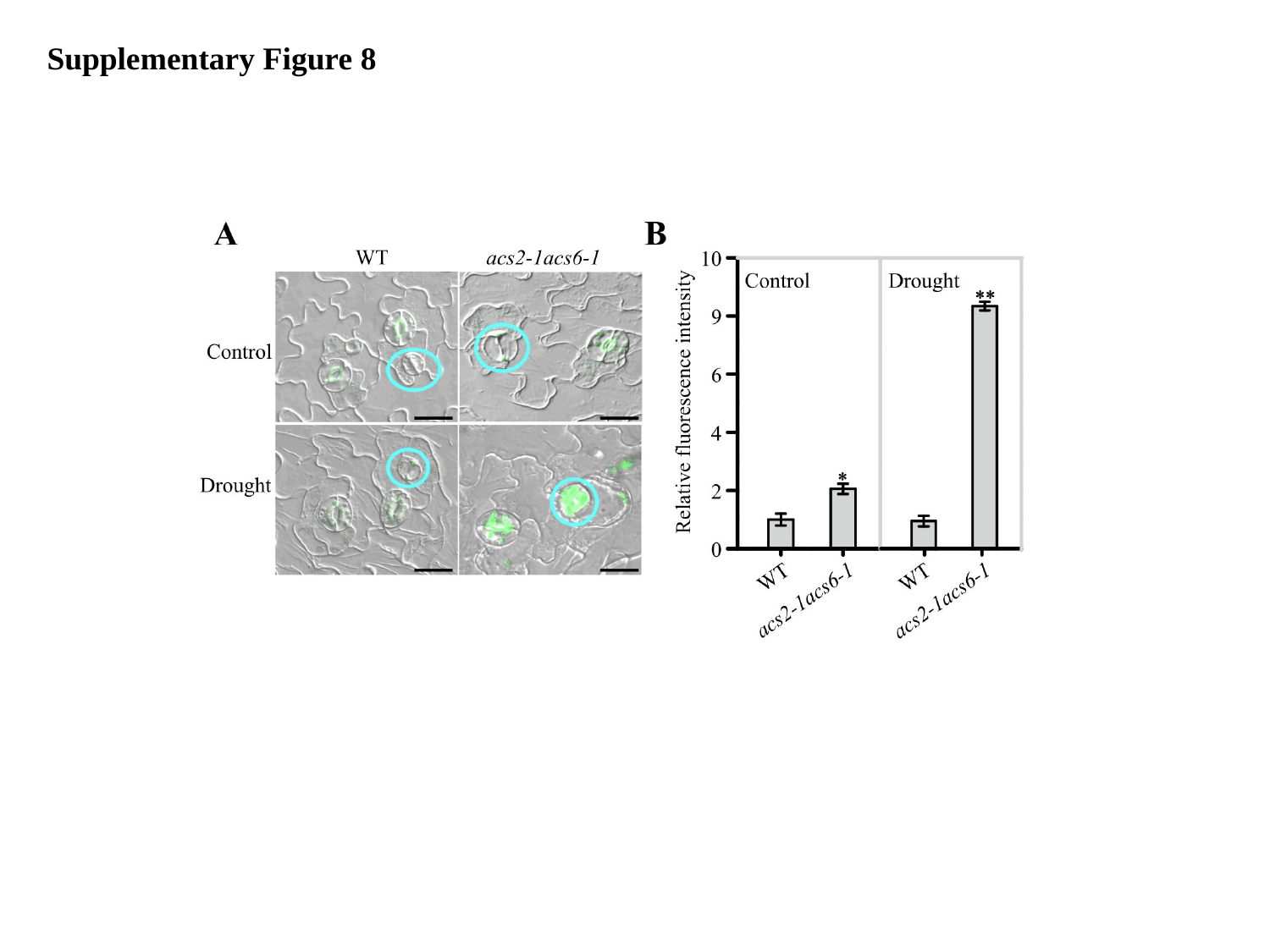

Supplementary Figure 8

## Slide 12
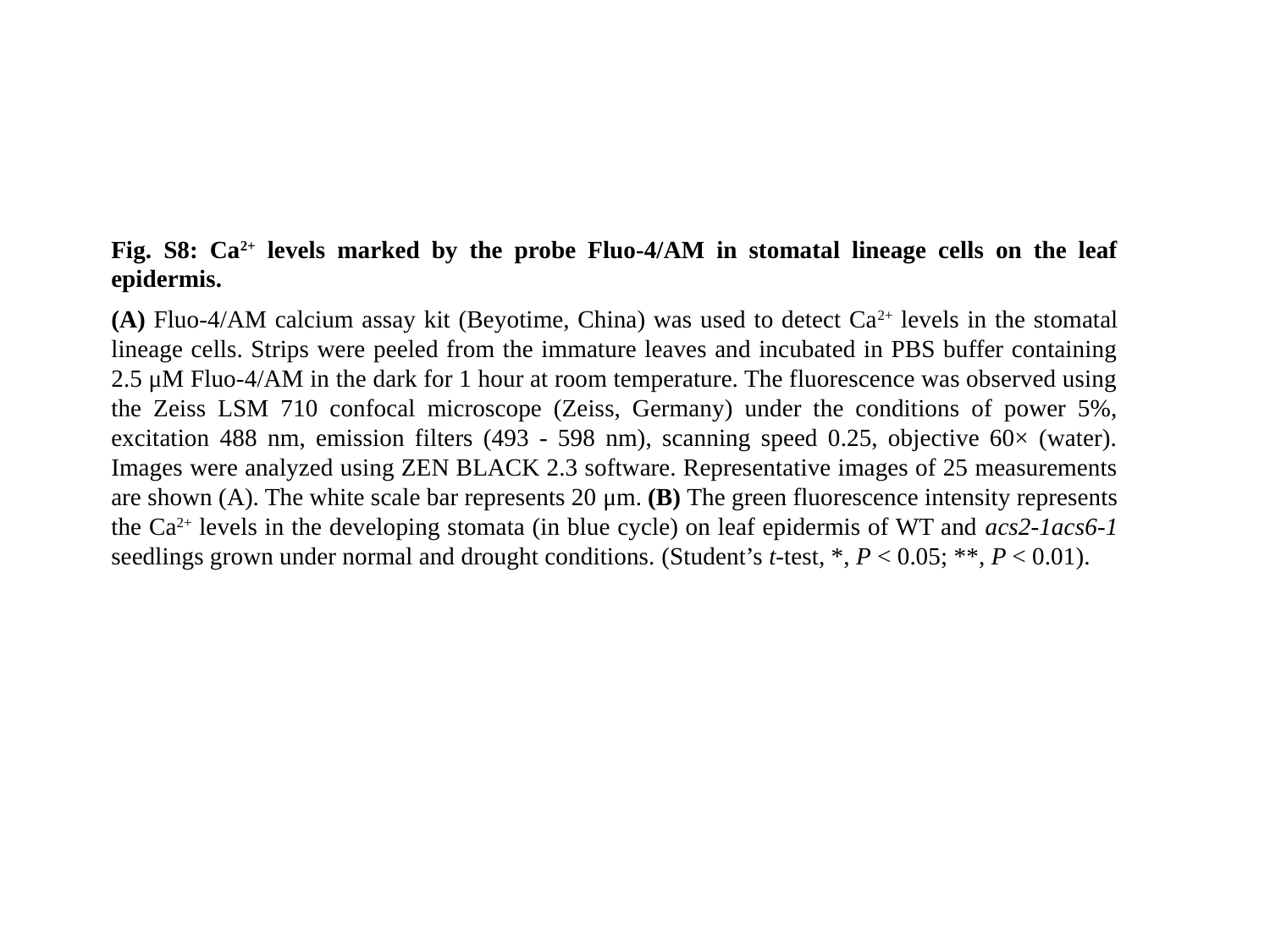

Fig. S8: Ca2+ levels marked by the probe Fluo-4/AM in stomatal lineage cells on the leaf epidermis.
(A) Fluo-4/AM calcium assay kit (Beyotime, China) was used to detect Ca2+ levels in the stomatal lineage cells. Strips were peeled from the immature leaves and incubated in PBS buffer containing 2.5 μM Fluo-4/AM in the dark for 1 hour at room temperature. The fluorescence was observed using the Zeiss LSM 710 confocal microscope (Zeiss, Germany) under the conditions of power 5%, excitation 488 nm, emission filters (493 - 598 nm), scanning speed 0.25, objective 60× (water). Images were analyzed using ZEN BLACK 2.3 software. Representative images of 25 measurements are shown (A). The white scale bar represents 20 μm. (B) The green fluorescence intensity represents the Ca2+ levels in the developing stomata (in blue cycle) on leaf epidermis of WT and acs2-1acs6-1 seedlings grown under normal and drought conditions. (Student’s t-test, *, P < 0.05; **, P < 0.01).
